# Quantifying Ligand Binding to the Surface of Metal–Organic Frameworks

**DOI:** 10.1021/jacs.3c04892

**Published:** 2023-07-24

**Authors:** Austin Wang, Kyle Barcus, Seth M. Cohen

**Affiliations:** Department of Chemistry and Biochemistry, University of California, San Diego, La Jolla, California 92093, United States

## Abstract

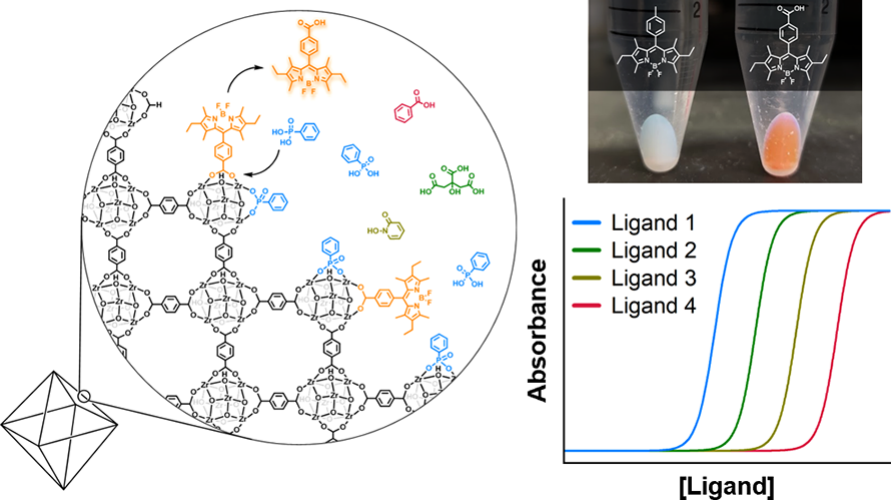

The binding of molecules to the exterior surface of metal–organic frameworks (MOFs) is not a well-understood phenomenon. Herein, the surface chemistry of three MOFs, UiO-66, MIL-88B-NH_2_, and ZIF-8, is investigated using dye-displacement experiments. MOF particle surfaces were modified with ligand-appended BODIPY dyes. The ability of the coordinated dyes to be displaced by a variety of exogenous ligands was measured by ultraviolet-visible spectroscopy. This method allowed for measurement of apparent binding constants for different ligands to the MOF surface. As might be expected, ligand affinity was dependent on the nature of the underlying metal–ligand composition of the MOF. This work provides a quantitative evaluation of ligand binding to MOF surfaces and important insights for the modulation, modification, and manipulation of MOFs.

## Introduction

Metal–organic frameworks (MOFs) are extended coordination solids that typically form as crystalline or microcrystalline particles with a variety of rich pore structures.^1^ The modification of the external surfaces of MOF via coordination chemistry is critical to many features of these materials, particularly the modulation of their nucleation/growth as well as the immobilization of small molecules or polymers for a variety of applications.^2−8^ Despite many studies that rely on the surface chemistry of MOFs, few studies have quantified these interactions.^9,10^ This is probably due, in part, to the porous nature of the MOF surface, which creates some ambiguity about where (interior vs exterior) and how molecules interact with the framework.

An early, inspiring study for the work reported here involved the attachment of ligand-appended 4,4-difluoro-4-bora-3a,4a-diaza-*s*-indacene (BODIPY) dyes to the surface of MOFs.^11^ A carboxylate-appended BODIPY dye was ligated to the surface of two pillared MOF structures, [Zn_2_(1,4-bdc)_2_(dabco)]_*n*_ and [Zn_2_(1,4-ndc)_2_(dabco)]_*n*_ (where 1,4-bdc, 1,4-ndc, and dabco are 1,4-benzenedicarboxylate, 1,4-naphthalenedicarboxylate, and 1,4-diazabicyclo[2.2.2]octane, respectively). These MOFs display a rectangular prism crystal habit, where four adjacent sides are terminated by carboxylate ligands and two opposite faces are terminated by dabco (nitrogen-based) ligands. When exposed to the carboxylate-BODIPY dye, the dye was shown by confocal microscopy to bind (presumably via ligand exchange) to the four [100] carboxylate-terminated faces. This study also showed BODIPY binding to the surfaces of [Cu_3_(1,3,5-btc)_2_}]_*n*_ (HKUST-1, 1,3,5-btc = 1,3,5-benzenetricarboxylate) as well as demonstrating that BODIPY dyes lacking the carboxylate functionality were not bound to the MOF surfaces. Similarly, motivated by a desire to study the behavior of colloidal MOF particles, the assembly of [Zn(mim)_2_]_*n*_ (ZIF-8, mim = 2-methylimidazolate) particles was visualized by modifying the surface of these particles with an imidazole-modified BODIPY dye.^12,13^ Again, confocal microscopy showed that dye modification was limited to the surface of the particles and did not alter any key chemical characteristics of the MOFs.

In another study, a method for modifying the external surfaces of Zr(IV)-based MOFs with 1,2-dioleoyl-*sn*-glycero-3-phosphate (DOPA) was described.^14^ DOPA was selected because this phosphate-based ligand was expected to coordinate strongly to, but not degrade, the Zr(IV) secondary building units (SBUs) on the surface of [Zr_6_O_4_(OH)_4_(1,4-bdc)_6_]_*n*_ (UiO-66), [(Zr_6_O_4_(OH)_4_(1,4-bpdc)_6_]_*n*_ (UiO-67, 1,4-bpdc = 1,4-biphenyldicarboxylate), and [(Zr_6_O_4_(OH)_4_(4,4′-eddb)_6_]_*n*_ (BUT-30, 4,4′-eddb = 4,4′-(ethyne-1,2-diyl)dibenzoic acid). Upon surface functionalization with DOPA, these MOFs retained their high surface area (indicating only surface functionalization) and became dispersible as colloids in low polarity solvents. Importantly, inductively coupled plasma atomic emission spectroscopy (ICP-AES) and a dye-labeled version of DOPA were used to quantify the amount of DOPA on the surface of the MOFs. It was found that the amount of DOPA modification on the particles correlated with the surface density of the SBUs, with DOPA coverage following the trend UiO-66 > UiO-67 > BUT-30. Taken together, these excellent studies of ligand-directed surface modification of MOFs create the foundation for evaluating the binding affinities of different ligands to the surface of MOFs.

**Figure 1:**
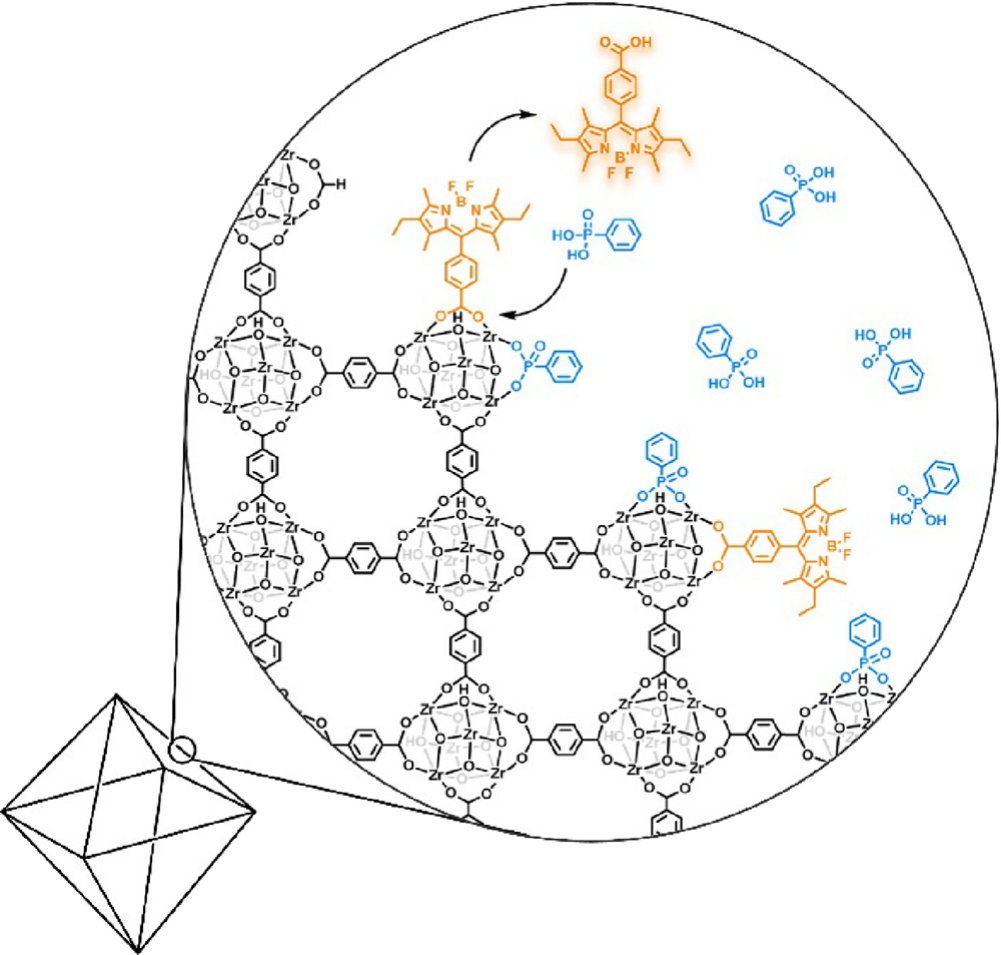
Conceptual illustration of surface exchange between coordinating ligands.

Herein, experiments were performed that provide apparent binding constants for various ligands to the surface of three MOF materials: UiO-66, MIL-88B-NH_2_, and ZIF-8. In these experiments, the MOF surface is treated as an extended coordination compound where the binding of ligands is directed to the metal ion nodes (i.e., SBUs; Figure 1). Carboxylate- or imidazole-appended BODIPY dyes were bound to the surface of the MOFs. By monitoring the displacement of these dyes by a range of small molecule ligands, apparent binding constants could be measured. Ligands showed clear differences in their ability to displace the bound dyes as a function of denticity, donor ability, and ligand compatibility with the SBU structure/composition. To the best of our knowledge, this represents the first quantitative study of ligand binding to the surface of MOFs. The observed trends suggest that basic principles of coordination chemistry provide a reasonable framework for conceptualizing the binding of these molecules to the surface of MOFs.

## Results and Discussion

Initial investigation into surface functionalization was conducted with UiO-66. UiO-66 nanoparticles were prepared using a previously reported method to prepare multigram quantities of monodisperse particles with a uniform size distribution and octahedral morphology as imaged by scanning electron microscopy (SEM; Figure S1).^15^ Powder X-ray diffraction (PXRD) of the recovered particles matched the simulated data of the crystal structure (Figures S2), and surface area measurements are consistent with previously reported values (Figure S3).^15^

**Figure 2:**
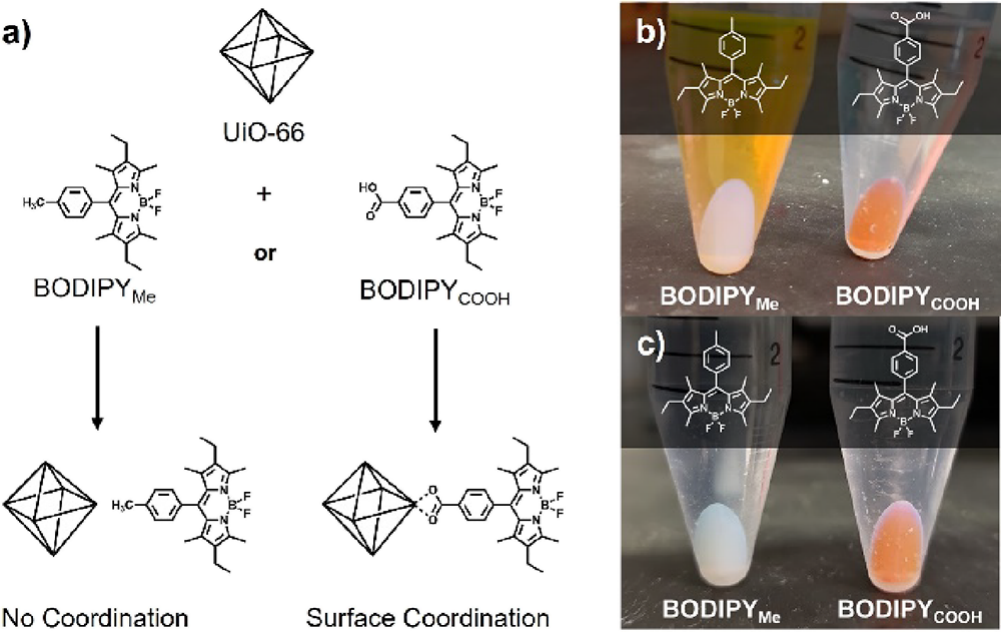
(a) Scheme of proposed dye interaction with UiO-66. (b) Image of UiO-66 particles 24 h after dye addition. (c) Image of isolated UiO-66 particles after multiple wash cycles with DMF.

To probe the surface binding of UiO-66, two dyes were prepared, 4,4-difluoro-8-(4-carboxyphenyl)-1,3,5,7-tetramethyl-2,6-diethyl-4-boron-3a,4a-diaza-*s*-indacene (BODIPY_COOH_),^16^ which contains a carboxylate group capable of coordinating the zirconium cluster of UiO-66, and 2,6-diethyl-4,4-difluoro-1,3,5,7-tetramethyl-8-(4-methylphenyl)-4-bora-3a,4a-diaza-*s*-indacene (BODIPY_Me_), which was used as a non-coordinating control (Figure 2 and Scheme S1, see the Supporting Information for details). While many dyes could be used for MOF surface binding, BODIPY_COOH_ provided several advantages: it is soluble in most non-polar organic solvents, it is amendable to further chemical modification (see below), it is not readily photobleached, it has a high molar absorptivity (ε = 69,094 M^–1^ cm^–1^) that can be measured at low concentrations, and a λ_max_ of 524 nm is well beyond the absorbance of solvents and organic ligands that are used to construct the MOF (Figure S4).

Stock solutions of BODIPY_COOH_ and BODIPY_Me_ were prepared in DMF at a concentration of 4 μM, and 1 mL of the dye stock solution was added to separate suspensions of 20 mg of UiO-66 in 1 mL of DMF (Figure 2). The solutions were vigorously mixed and left to stand overnight, after which the particles were collected by centrifugation and thoroughly washed with DMF to remove any residual dye. Photographs of the samples after the initial centrifugation show a clear difference between the two samples (Figure 2b,c). The supernatant of the particles treated with BODIPY_Me_ was colored, and the dye was easily removed from the MOF after washing with DMF. By contrast, the supernatant of the particles treated with BODIPY_COOH_ was colorless while the UiO-66 particles became a bright orange color that persisted even after extensive washing. These results suggest that dye coordination is occurring at the SBUs and that the dye was not bound to the MOF by weak, non-covalent interactions to the crystal surfaces or trapping of the dye in the pores of the MOF. The kinetics of BODIPY_COOH_ coordination to the surface of UiO-66 was also investigated. MOF particles were isolated, washed, and analyzed for 10 min, 1 h, and 24 h after the addition of the dye. Absorption measurements of the digested particles show that the surface modification is rapid, with ∼80% of the maximum dye coordination (based on coverage at 24 h) occurring within the first 10 min (Figure S5).

**Figure 3:**
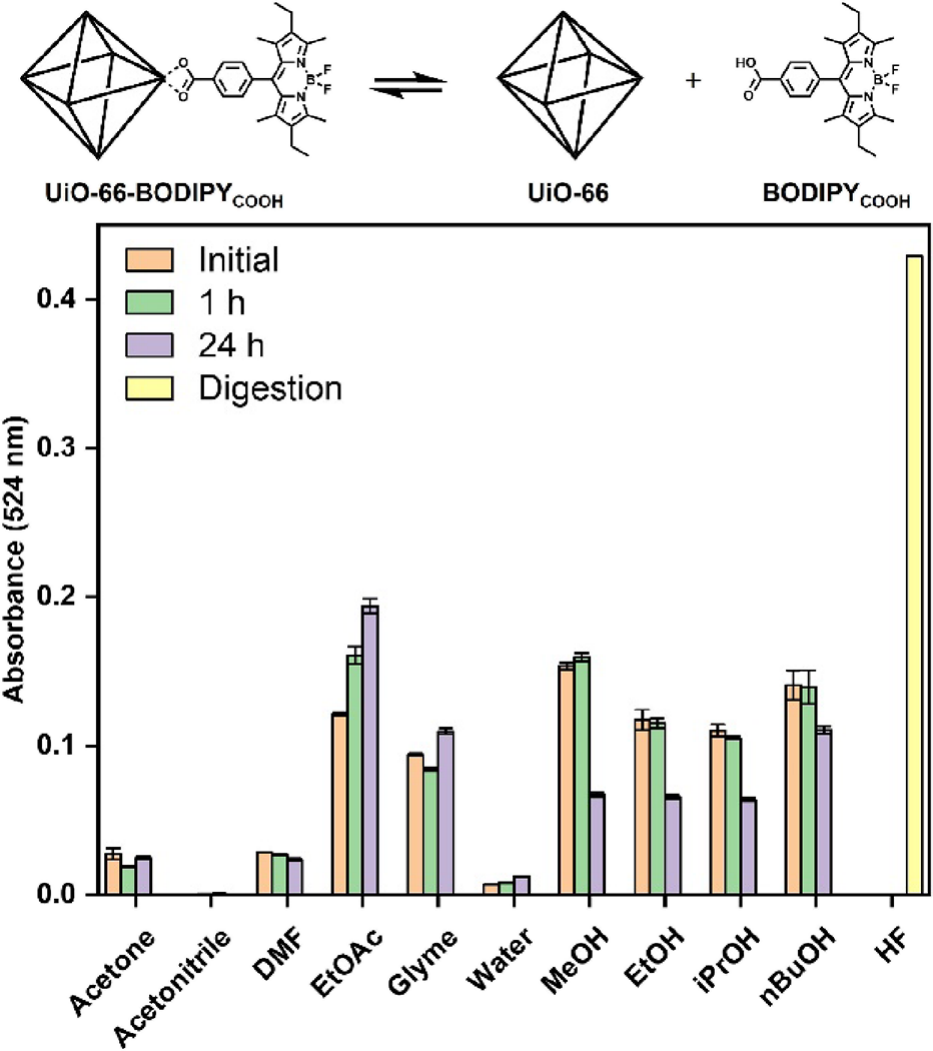
Solvent stability of BODIPY_COOH_ coordination to UiO-66. After centrifugation at each timepoint, UV–visible spectroscopy measurements of the supernatant at 524 nm were used to determine the amount of dye removed from the MOF surface.

While the BODIPY_COOH_ could not be easily washed from the MOF surface, it was observed that if UiO-66-BODIPY_COOH_ was suspended in DMF for 24 h, then the supernatant solution gradually became colored over time, indicating dissociation of the dye from the MOF surface. Having observed this, the effect of different solvents on the stability of dye coordination and rate of dye removal was tested by examining the amount of dye present in the supernatant of UiO-66-BODIPY_COOH_ particles after dispersion (Figure 3). The particles were first dispersed in different solvents and then pelleted by centrifugation at different timepoints: immediately after dispersing (*t* = 0), 1 h, and 24 h, after which the supernatant was removed to determine the concentration of the dye by UV–visible spectroscopy. It should be noted that all solvents were standard ACS grade, used as received, and no special precautions were taken to protect the solvents from the atmosphere. The results show that the solvent plays a significant role in the stability of dye coordination, with acetone, acetonitrile, and DMF resulting in the lowest degree of dye removal. Surprisingly, water, which many MOFs are not structurally stable in, was ineffective at removing the dye. This is most likely due to the low water solubility of the dye, which inhibits dissociation from the MOF surface.

For alcohol solvents, an initial, relatively large dye displacement was observed (at *t* = 0 and 1 h), but then, the concentration of the dye in solution decreased at 24 h (relative to the 1 h timepoint). With the exception of nBuOH, the concentration of the dye in MeOH, EtOH, and iPrOH all equilibrated to nearly the same value. While the reason behind this phenomenon is not fully understood, MeOH is widely used as an activation solvent for UiO-66 due to its distinct ability to remove or exchange loosely coordinated ligands and modulator from the MOF interior.^17^ One hypothesis is that MeOH, and to a lesser extent EtOH and iPrOH, facilitates reversible ligand-binding of both the monotopic dye and the ditopic framework ligands (i.e., 1,4-H_2_bdc) near the surface. The initial rapid dissociation of the dye is followed by a gradual dissociation of 1,4-H_2_bdc. Both the dye and 1,4-H_2_bdc compete for the newly exposed open metal sites, and the larger steric size and hydrophobicity of the dye may shift the surface coordination equilibrium favorably toward the dye.

Overall, the data in Figure 3 show that UiO-66-BODIPY_COOH_ coordination is most stable in polar, aprotic solvents such as acetone, acetonitrile, and DMF, while nonpolar and polar protic solvents lead to dye loss over time. The higher stability of the coordination in polar aprotic solvents over polar protic solvents suggests that a proton source may facilitate ligand exchange via protonation of the carboxylic acid on the coordinating dye. A similar mechanism was proposed and verified computationally in a previously reported study.^18^ As no attempt was made to rigorously exclude water from the solvents, ligand displacement could occur in aprotic solvents as well. However, the reason for the low dye stability in nonpolar, aprotic EtOAc and glyme is unclear. It may be that the weak metal-coordinating ability of these solvents could manifest as dye loss when present in high concentrations (i.e., as a solvent).

Based on the solvent stability (Figure 3), acetonitrile and acetone were considered the best solvents for titration experiments. Acetonitrile proved ineffective at solubilizing several of the compounds used in subsequent experiments (see below); therefore, acetone was used for all further experiments to minimize solvent effects on dye coordination.

**Figure 4:**
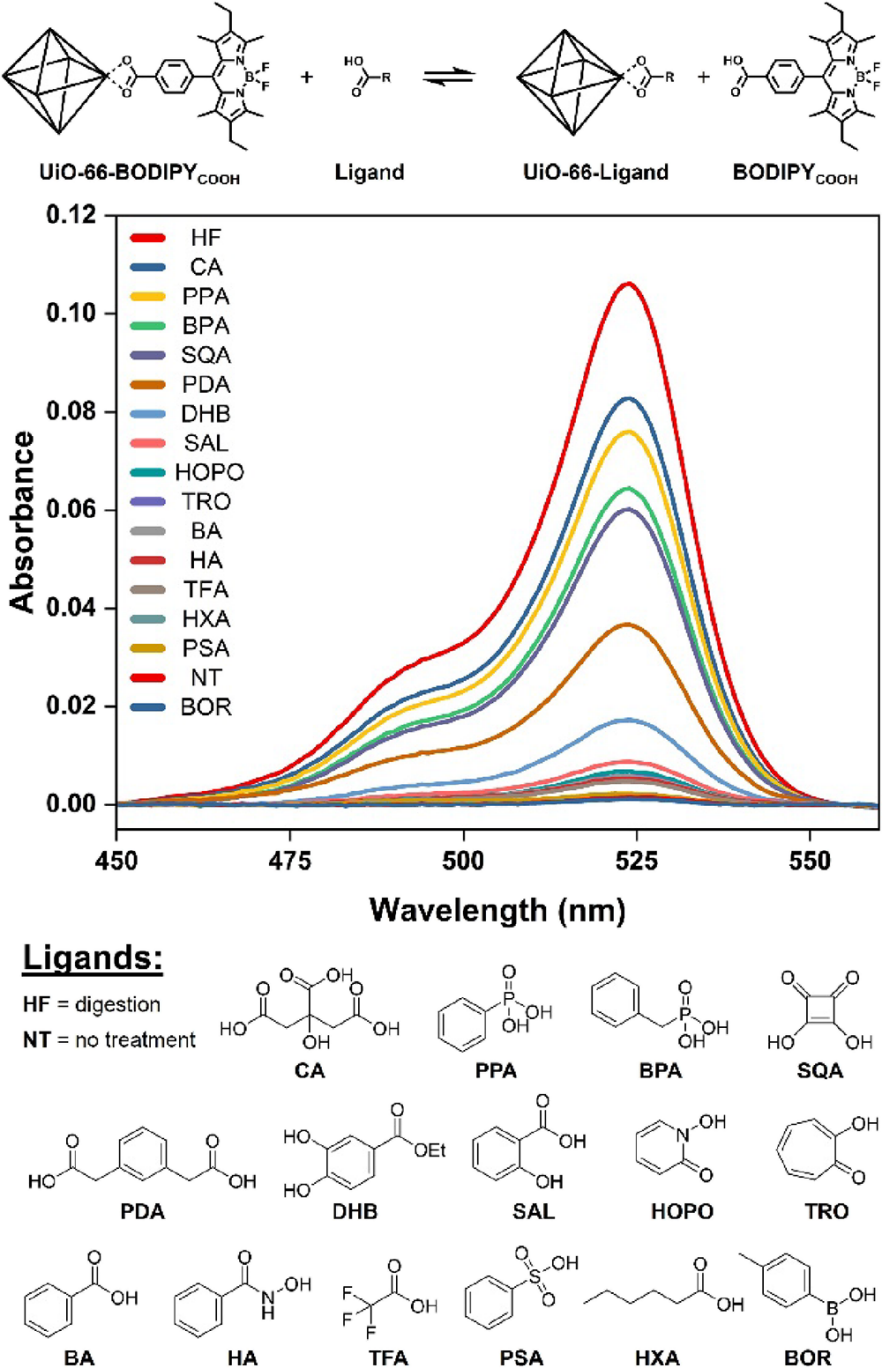
UV–visible spectra of UiO-66-BODIPY_COOH_ solution supernatants after treating with various ligands. Greater absorbance corresponds to stronger ligand binding affinity (i.e., greater dye displacement).

Having established a suitable solvent system to study ligand binding, the inherent reversibility of BODIPY_COOH_ coordination to the surface of UiO-66 was used as a tool to measure the relative binding affinity of a variety of ligands to UiO-66. To achieve this, an initial screening of ligands was performed to determine the general structural and chemical features that result in dye displacement (Figure 4). The initial examination was designed to evaluate ligand binding at a single concentration. UiO-66-BODIPY_COOH_ in DMF was the first solvent exchanged with acetone and diluted to a concentration of 4 mg/mL. Individual Eppendorf tubes were prepared with 1 mL of the MOF suspension to which 10 μL of ligand stock solutions (prepared at 10 mM in acetone) was added (approximately a 62:1 ligand to surface dye ratio). The solutions were promptly mixed and left to equilibrate for 24 h, after which the particles were collected by centrifugation and the amount of dye displaced was determined by UV–visible spectroscopy measurements on the supernatant. The displacement by each ligand was then compared relative to two control samples of UiO-66-BODIPY_COOH_ with either no treatment (**NT**) or complete ligand displacement by digestion of the MOF using HF (labeled **HF**; Figure 2, Figure 4).

Simple carboxylic acid ligands of different acidities (**BA**, **HXA**, and **TFA**) showed only a small amount of dye displacement in the single concentration experiment. However, multitopic carboxylic acids showed a very high binding affinity, with the tritopic citric acid (**CA**) providing the highest relative dye displacement of all the ligands tested. Phosphonates and phosphates are well known for their ability to form strong coordination bonds to metal oxides, which has led them to be widely used in the surface functionalization of many materials, including UiO-66 and zirconium oxide nanoparticles.^6,14,19^ Their affinity was confirmed here as both the phenylphosphonic acid (**PPA**) and benzylphosphonic acid (**BPA**) were comparable in binding strength to **CA** and stronger than ditopic carboxylic acid (**PDA**). In contrast, hydroxamic acid (**HA**), 2-hydroxypyridine *N*-oxide (**HOPO**), and catechol (**DHB**), all ligands also known for strong metal chelation to hard Lewis acids like Zr(IV), were relatively poor ligands, resulting in only slightly higher dye displacement than the monotopic carboxylic acids. The weak binding of these ligands when compared to the phosphonates demonstrates the value of this methodology as it allows for the relative binding strength of different ligands to be quickly confirmed and in doing so provide insight for the similarities and differences in MOF surface chemistry when compared to classical coordination complexes based on the same metal ions.

Interestingly, while ligand p*K*_a_ could contribute to dye displacement by simple protonation of the carboxylate ligand of the BODIPY dye, the results in Figure 4 indicate that the strength of the resulting metal–ligand bond is more important than acidity. This can be illustrated by comparing the high dye displacement of the phosphonic acids (**PPA** and **BPA**, p*K*_a_ = 1.2 and 1.8, respectively) when compared to the negligible binding of phenylsulfonic acid (**PSA**, p*K*_a_ = 1.4). The negligible role of ligand acidity is further supported by the comparable dye displacement effect obtained with trifluoroacetic acid (**TFA**, p*K*_a_ = 0.3), benzoic acid (**BA**, p*K*_a_ = 4.2), and hexanoic acid (**HXA**, p*K*_a_ = 5.0). However, it should be noted that these p*K*_a_ values are measured in water while the experiments performed here are reported in acetone. As such, the true p*K*_a_ of these ligands is unknown and the effect of acidity cannot be unambiguously confirmed by these results alone. While experiments in water would be of interest, the insolubility of the BODIPY_COOH_ dye used in these studies prevents these titrations under the current experimental conditions.

**Figure 5:**
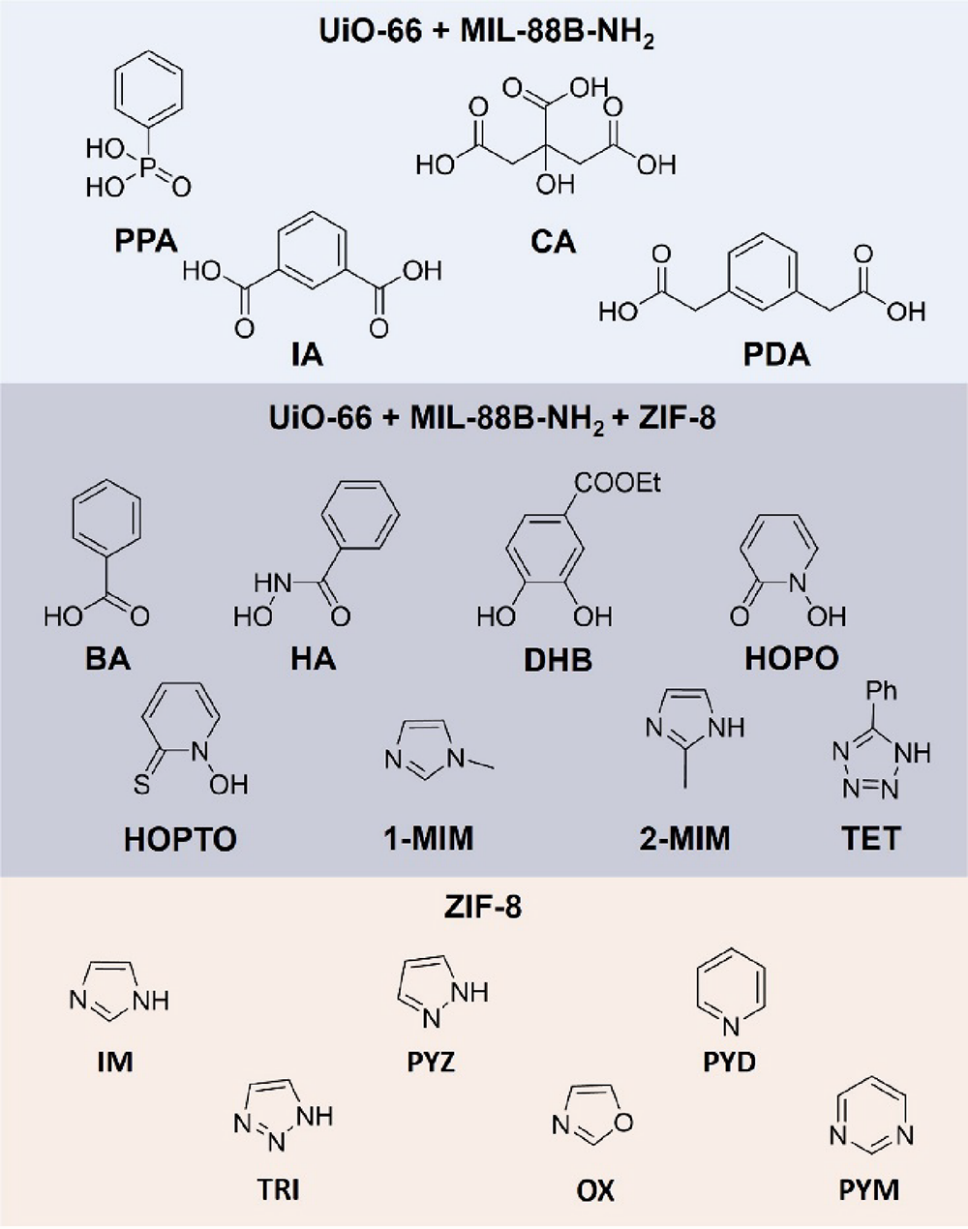
Ligands used for competitive binding titration experiments and the dye-coordinated MOFs they were tested with. Ligands in the middle box were used for all MOFs in the study for direct comparison.

To get a more refined comparison of the relative ligand affinities for UiO-66, a subset of the ligands (Figure 5) was selected for additional titration experiments where dye displacement was monitored as a function of the ligand concentration.

**Figure 6:**
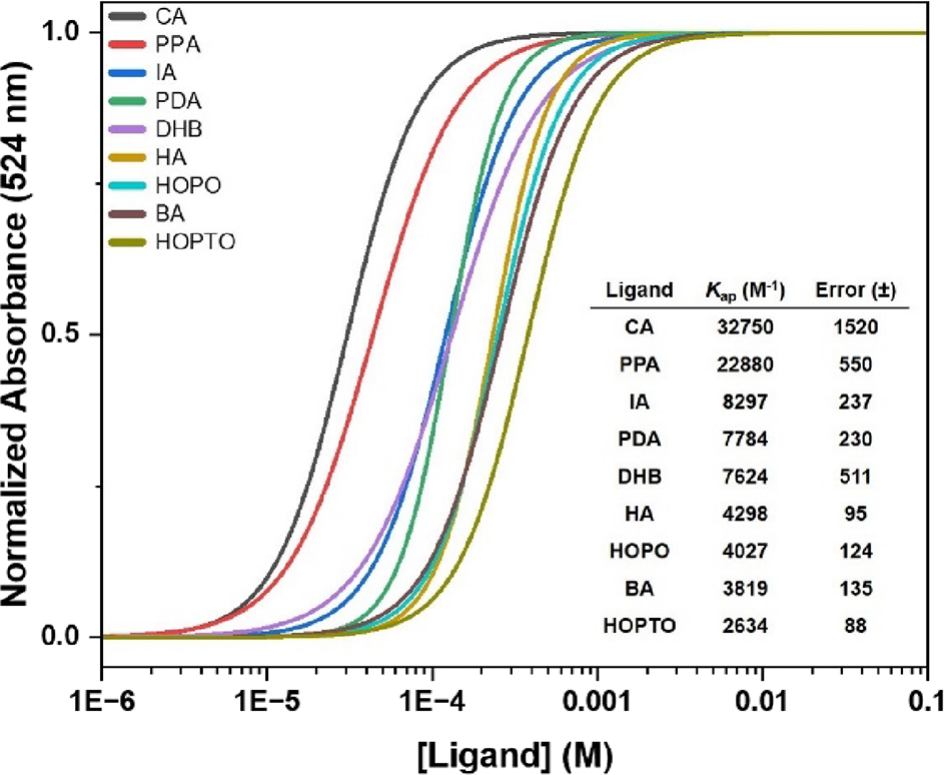
Normalized titration curves of UiO-66-BODIPY_COOH_ with increasing concentrations of select ligands (Figure 5, top and middle). The inset table gives the ligand, apparent binding constant (*K*_ap_), and error values.

To determine the apparent binding constants of each ligand, the same experimental setup as performed for the single-point screen was used. In this case, the concentration range studied was prepared by serial dilutions of ligand stock solutions and each concentration was measured in triplicate. The absorption of the supernatant at each concentration was then plotted as a function of the concentration, and the points were fit with a sigmoidal curve (Figures S6 and S7, see the Supporting Information for details). The concentration at the inflection point of the curve for each ligand was found, and the inverse of this concentration is taken as the apparent binding constant (inset table; Figure 6). It should be emphasized that, for ease of visual comparison, the curves from the sigmoidal fits shown in Figure 6 have been normalized; however, the values for the apparent binding constant are taken directly from the original raw titration data (Figure S7). As expected, the titrations show that the trends in apparent ligand affinity follow the results of the single-point experiments (Figure 4). Citric acid (**CA**) and phenylphosphonic acid (**PPA**) show apparent binding constants (*K*_ap_) 4× and 3× larger than the next best ligand, respectively. However, the relative difference in affinity between benzoic acid (**BA**) and the ditopic carboxylic acids (e.g., **PDA** and **IA**) is modest, being slightly greater than 2× (see the table inset in Figure 6). The amine heterocycles **2-MIM**, **1-MEM**, and **TET** were far weaker ligands, with estimated binding affinity values that are very poor and could only be estimated by the incomplete titration data (>1 M; Figure S7). To ensure that dye displacement was not a result of MOF degradation, SEM imaging was performed on particles post-treatment. Apart from **PPA** and **HOPO**, the structure of UiO-66 remained unchanged for all ligands throughout the concentration range tested (Figures S8 and S9). For **PPA** and **HOPO**, significant restructuring or total dissolution of the UiO-66 particles was observed at the highest concentrations (Figure S10). However, no visible change to the MOF particles was observed at concentrations along the inflection points of the titration curves was noted (Figure 6), indicating that the structure of the MOF remains unchanged.

Overall, these data are valuable for identifying ligands that might be best suited to functionalize an MOF particle or modulate MOF growth under select reaction conditions. To confirm this, BODIPY_COOH_ was modified with a phosphonic acid group using standard amide coupling to form BODIPY_PHOS_ (see the Supporting Information for details). Given the much higher binding affinity of the phosphonate group over carboxylate, BODIPY_PHOS_ was expected to show both greater solvent stability and require significantly higher concentrations of competitive ligands to displace from the MOF surface. Indeed, BODIPY_PHOS_ coordination to UiO-66 was far more stable, with only small amounts of displacement occurring in alcohols and negligible displacement in all other solvents (Figure S11). Competitive binding experiments were even more compelling, where examination of a subset of ligands showed that none of the ligands were capable of displacing the BODIPY_PHOS_ except **PPA** and to a much lesser extent **CA** and **HA** (Figure S12). In the case of **PPA**, the concentrations at which dye displacement was detected were above the point at which MOF degradation occurs. This highlights the role of coordination chemistry in MOF surface modification and that with the selection of a strong ligand, functionalization of the MOF can be considered extremely stable (on par with MOF stability).

**Figure 7:**
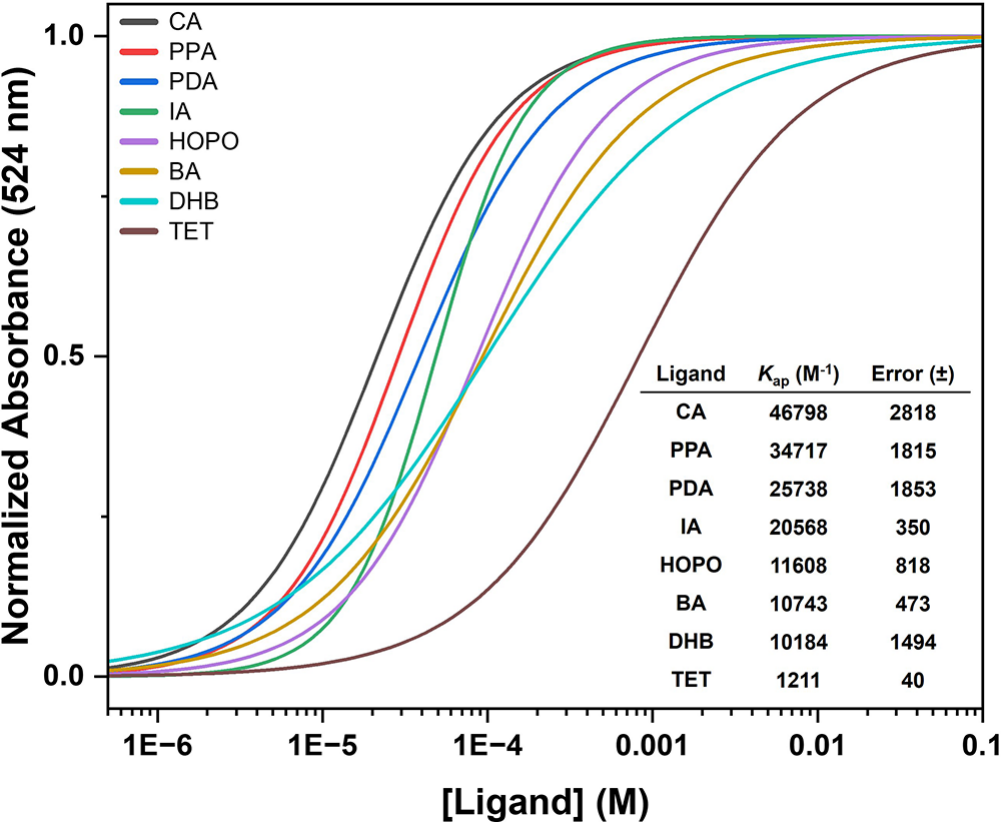
Normalized titration curves of MIL-88B-NH_2_-BODIPY_COOH_ with increasing concentrations of select ligands (Figure 5, middle and bottom). The inset table gives the ligand, apparent binding constant (*K*_ap_), and error values.

The same methodology described for UiO-66 was applied to study ligand binding to MIL-88B-NH_2_, an MOF composed of trimeric Fe(III) SBUs and amino-terephthalic acid linkers (H_2_bdc-NH_2_) with a hexagonal rod morphology (Figures S13 and S14).^20^ After functionalization with BODIPY_COOH_, the solvent stability procedure was repeated using the same solvents as were used for UiO-66 (Figure S16) and concentration-dependent ligand binding (Figure 7) was performed using the same ligand set as UiO-66 (Figure 5) in acetone. The ligands 2-mercaptopyridine *N*-oxide (**HOPTO**) and hydroxamic acid (**HA**) partially dissolved this MOF at high concentrations forming brightly colored complexes (i.e., resulting in the deeply colored Fe(**HOPTO**)_3_ complex when the titration was attempted with **HOPTO**), making determination of apparent binding affinities impossible with these compounds. When comparing the results of the MIL-88B-NH_2_ to UiO-66, the order of ligand strength is similar, with both **CA** and **PPA** remaining the tightest binding ligands. However, the absolute values for the apparent binding constant of the ligands are much higher in the case of MIL-88B-NH_2_. This is possibly due to the weaker coordination of carboxylates to Fe(III) over Zr(IV). SEM images of the particles after ligand treatment showed that MIL-88B-NH_2_ was similarly stable to ligand treatment at relevant concentrations (Figure S18). Degradation was most apparent with **DHB** and **HOPO** but only at concentrations well above complete dye displacement (Figure S19).

Finally, zeolitic imidazolate framework 8 (ZIF-8) was synthesized and examined for dye displacement.^21,22^ ZIF-8 is composed of individual Zn(II) ions bridged by 2-methylimidazole ligands. The binding strength of carboxylate ligands to the mononuclear Zn(II) SBUs is weaker than imidazole ligands, as reasoned by hard-soft Lewis acid–base theory. As the purpose of this study was to determine the strength of ligand binding to the surface of the native MOF, the BODIPY_COOH_ dye was modified with histamine using standard amide coupling methods to form BODIPY_Im_ (see the Supporting Information for details). The same process for dye coordination, solvent stability, ligand screening, and ligand titrations was performed on ZIF-8 (Figures S23 and S24).

Six heterocycles were chosen for the titration experiments, including eight that were used for UiO-66 (Figure 5, middle and bottom). The curve fittings from the titration and the corresponding *K*_ap_ values are shown in Figure S24 and Table S1. Unlike UiO-66 and MIL-88B-NH_2_, most of the ligands showed very weak binding to ZIF-8. Additionally, SEM imaging of the particles after titration with the two strongest ligands **HOPO** and **TET** clearly shows that the observed dye displacement is a result of particle degradation, although the particles were remarkably stable to more basic ligands such as 2-methylimidazole (**2-MIM**) and imidazole (**IM**) (Figure S25). As a result of the weak binding, none of the ligands tested displaced the BODIPY_Im_ dye to a significant value within the concentration range tested. While this precludes the determination of accurate apparent binding constants (values in Table S1 are best fits based on incomplete titrations), many of the titration curves show sufficient differences that some general observations can be made for ZIF-8. The binding of ligands to ZIF-8 shows a clear preference for five-membered heterocycles when compared to similar six-membered rings. This is clearly demonstrated by the strongly binding imidazoles (**IM** and **2-MIM**) by comparison to pyrimidine (**PYM**), despite having the same 1,3-*N*,*N* donor arrangement. In addition, the ability of the heterocycle to act as a bidentate bridging ligand also appears important. Five-membered rings with only a single amine available for binding, such as *N*-methyl imidazole (**1-MIM**) and oxazole (**OX**), are among the weakest ligands for ZIF-8 (Figure S24 and Table S1). These results suggest that the binding by BODIPY_Im_ to the ZIF-8 surface likely occurs via a bridging coordination mode using both nitrogen donor atoms, and as such, dye displacement requires a similarly strong binding mode to displace the dye from the surface. SEM imaging of the particles after treatment with many ligands also shows large changes in ZIF-8 morphology, indicative of MOF restructuring or degradation (Figure S25). In the case of **TET** at 14.3 mM, the images show the formation of much larger particles with a distinctly hollow, intergrown morphology. While extensive characterization was not performed, PXRD of the recovered solid indicates that the particles remain crystalline but are not ZIF-8 (Figure S26).

In conclusion, a simple methodology to measure and quantify the relative binding strength and apparent binding constant of ligands to the surface of MOFs has been developed. By first coordinating a BODIPY dye to the surface of the MOF, the addition of exogenous ligands to compete with the dye for coordination at the MOF surface allows for a means to measure relative binding constants. In this first report, UiO-66, MIL-88B-NH_2_, and ZIF-8 were examined as test cases with more than a dozen ligands. With the surface modification of MOFs becoming increasingly important for the field, the methods described here should help advance the understanding and manipulation of MOF surfaces and aid in efforts to optimize conditions for MOF surface modulation and functionalization. Importantly, the findings here suggest that the MOF surface can be considered to behave much like an extended coordination compound, which lends itself to rational design and selection of surface modifying groups.

## Supporting Information Available

Synthesis and characterization details for all compounds, dye functionalization and titration experimental details, data fitting procedure, Scheme S1, Table S1, and Figures S1–S26 (PDF)
