# Supplementary material for: Quantifying Ligand Binding to the Surface of Metal–Organic Frameworks
Source: J Am Chem Soc. 2023 Jul 24;145(30):16821–7. doi: 10.1021/jacs.3c04892 (PMC10401703; doi:10.1021/jacs.3c04892)
Supplement: Supplementary file 1 — ja3c04892_si_001.pdf [file ja3c04892_si_001.pdf]

Quantifying Ligand Binding to the Surface of Metal-Organic Frameworks

Austin Wang, Kyle Barcus, and Seth M. Cohen*

Department of Chemistry and Biochemistry, University of California, San Diego, La Jolla, California 92093, United States

SUPPORTING INFORMATION

Materials

All solvents were purchased from either Millipore Sigma or Fisher Scientific of ACS Grade (< 0.2% H_2O minimum) and used as received. No special precautions were taken to prevent exposure to atmospheric moisture. 3-Ethyl-2,4-dimethylpyrrole (97%) was purchased from TCI and monomethyl terephthalate was purchased from Combi-Blocks and used as received.

Characterization

Nuclear Magnetic Resonance. Proton nuclear magnetic resonance spectra (^{1}H NMR) were recorded on a JEOL ECA 500 spectrometer (500 MHz). Chemical shifts are reported in parts per million (ppm) referenced to the appropriate solvent peak.

Powder X-Ray Diffraction (PXRD). Dry MOF powder (~50 mg) was loaded into a small-well steel sample holder. PXRD was collected at ambient temperature on a Bruker D8 Advance diffractometer at 40 kV, 40 mA for Cu $K\alpha$ (l = 1.5418 Å), with a scan speed of 2 sec/step, a step size of 0.05° in 2θ, and a 2θ range of 2-50°.

Scanning Electron Microscopy (SEM). MOF particles were suspended in acetone (~1 mg/mL) and spotted onto silicon wafers using a thin glass capillary. The silicon wafers were mounted on an aluminum sample holder disk with carbon tape and coated using an Ir-sputter coating. A FEI Apreo SEM instrument was used for acquiring images using an accelerating voltage of 5 kV under vacuum at a working distance at 10.0 mm.

BET Surface Area Analysis. Samples for analysis were evacuated on a vacuum line overnight at room temperature prior to analysis. Samples (~50 mg) were then transferred to pre-weighed sample tubes and degassed at 105 °C on a Micromeritics Smart VacPrep for a minimum of 12 h or until the outgas rate was <5 mmHg. After degassing, the sample tubes were re-weighed to obtain a consistent mass for the samples. BET surface area (m^2/g) measurements were collected at 77 K with N_2 on a Micromeritics Tristar II Adsorption Analyzer using volumetric techniques.

UV-visible Spectroscopy. Solutions were prepared in the indicated solvent and 1 mL was transferred to a 1.5 mL semi-micro cuvette with a 1 cm path length. An Agilent Cary 60 was used to acquire spectra with a scan rate 2400 nm/s. Molar absorption coefficients were determined from serial dilutions of a stock solution and repeated in triplicate. Cuvettes were purchased from Brandtech Scientific (cat. no. 759165) to ensure compatibility with organic solvents such as acetone and DMF.

Synthesis of BODIPY$_{COOH}$

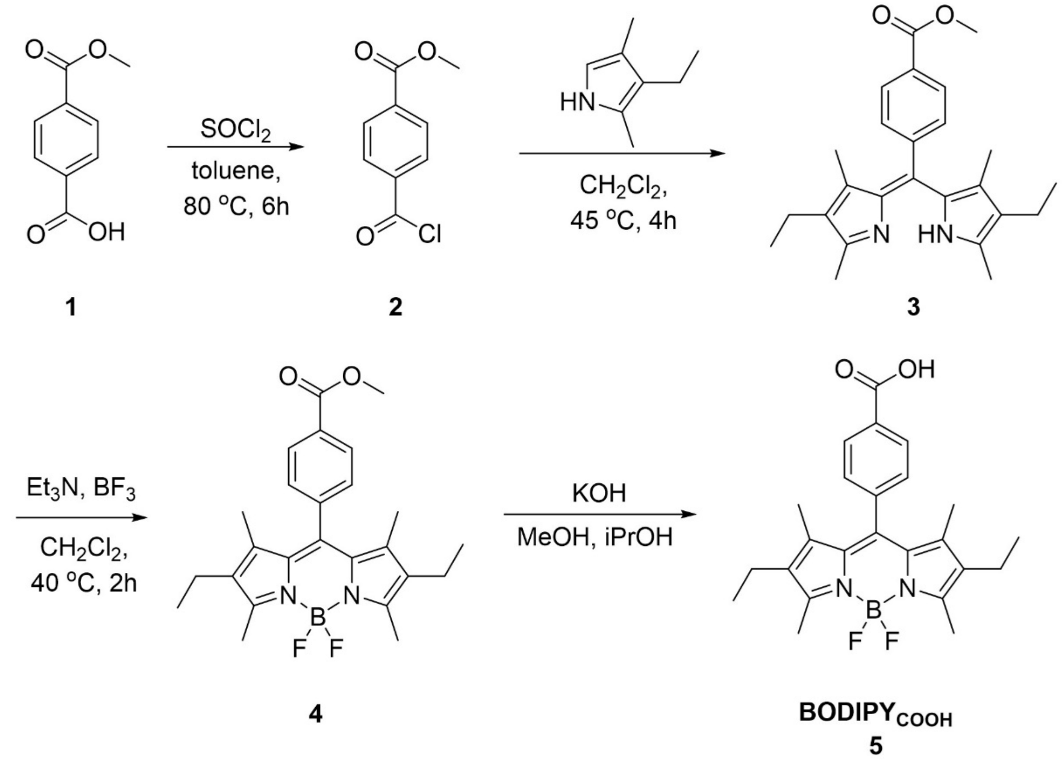

Scheme S1. Synthesis of 4-(4,4-difluoro-1,3,5,7-tetramethyl-3a,4a-diaza-4-bora-*s*-indacen-8-yl)benzoic acid **(5)**.

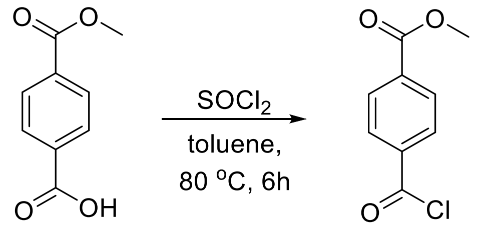

1 **2**

Methyl 4-(chlorocarbonyl)benzoate (2). A 500 mL round bottom flask was charged with monomethyl terephthalate (**1**, 25.0 g, 139 mmol, 1 eq.) and 100 mL of toluene. Thionyl chloride (12.2 mL, 167 mmol, 1.2 eq.) and 100 µL DMF was added and the suspension was stirred for 6 h at 80 °C during which the material completely dissolved. The reaction

was evaporated to dryness under vacuum and residual thionyl chloride was removed by co-evaporation with dry toluene (2×50 ml) to yield a white solid. The crude product was used for the next reaction without further purification.

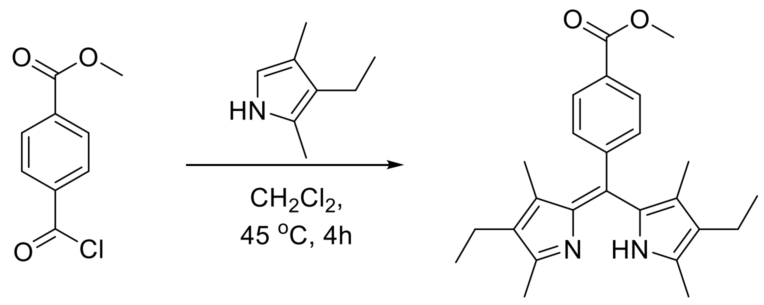

2 **3**

Methyl (*Z*)-4-((4-ethyl-3,5-dimethyl-1*H*-pyrrol-2-yl)(4-ethyl-3,5-dimethyl-2H-pyrrol-2-ylidene)methyl)benzoate (3). The crude acid chloride (**2**) from the previous step (19.0 g, 96 mmol, 2.4 eq.) was directly transferred to a 500 mL round bottom under Ar and 200 mL of dry CH_2Cl_2 was added via cannula. 3-Ethyl-2,4-dimethyl pyrrole (5.48 mL, 41 mmol, 1 eq.) from a fresh ampule was added via gas-tight syringe dropwise. The reaction mixture was heated to 45 °C for 4 h, after which the reaction mixture was evaporated to dryness under vacuum. The crude product was dry loaded on silica and purified by column chromatography (EtOAc:Hexane 0-70%, elutes at 30%) to yield a red solid which was used directly in the next step without further characterization due to its low stability. Yield: 5.30 g (67%).

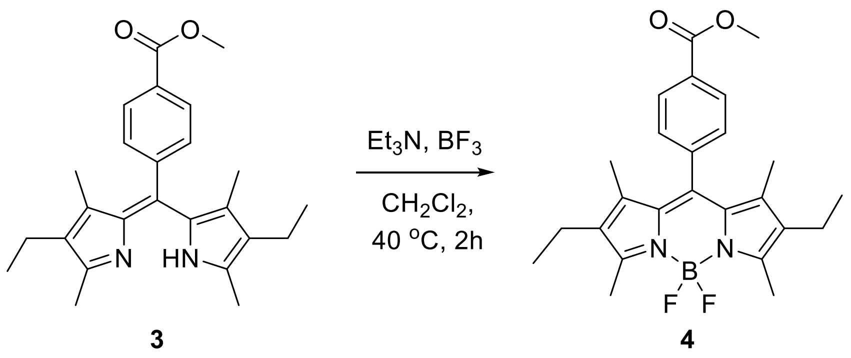

Methyl 4-(4,4-difluoro-1,3,5,7-tetramethyl-3a,4a-diaza-4-bora-*s*-indacen-8-yl)benzoate (4). The crude dipyrromethene (**4**, 5.00 g, 12.8 mmol, 1 eq.) was dissolved in anhydrous CH_2Cl_2 and freshly distilled Et_3N (8.92 mL, 64 mmol, 5 eq.) was added under argon. After stirring for 30 min at room temperature $BF_3{\cdot}Et_2O$ (16.2 mL, 128 mmol, 10 eq.) was added dropwise via syringe. The reaction mixture was then stirred at 40 °C until TLC showed complete conversion (~2 h). The reaction was cooled to room temperature and quenched with water. The CH_2Cl_2 layer was washed repeatedly with water (3×100ml), saturated sodium bicarbonate (1×100 ml), and brine then dried with sodium sulfate, filtered, and evaporated under vacuum. The crude material was dry loaded on silica and purified by column chromatography (EtOAc:Hexane 0-100%) and the product fractions were combined, evaporated under vacuum, and recrystallized from MeOH to give (**4**) as large red crystals. Yield: 3.28 g (59%). ^1H NMR (500 MHz, $CDCl_3$): *δ* 8.17 (d, *J* = 8.2 Hz, 2H), 7.40 (d, *J* = 8.0 Hz, 2H), 3.98 (s, 3H), 2.53 (s, 6H), 2.30 (q, *J* = 7.5 Hz, 4H), 1.27 (s, 6H), 0.98 (t, *J* = 7.5 Hz, 6H) ppm. ESI-MS(+) *m*/*z* calculated for: $[C_{25}H_{29}BF_2N_2O_2+H]^+$: 439.24, found: 439.35.

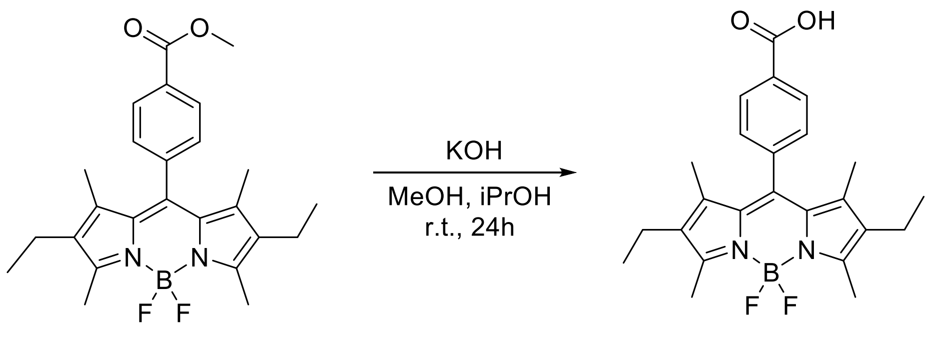

4 **BODIPY$_{COOH}$** **5**

4-(4,4-Difluoro-1,3,5,7-tetramethyl-3a,4a-diaza-4-bora-*s*-indacen-8-yl)benzoic acid (5). BODIPY$_{COOMe}$ **(4)** (500 mg, 1.14 mmol, 1 eq.) was first dissolved in minimal THF. After complete dissolution 50 mL of iPrOH was added and fully mixed. An aqueous solution of KOH (256 mg, 4.56 mmol, 4 eq.) dissolved in 50 mL of water was added. The suspension was monitored until TLC confirmed disappearance of the starting material. The reaction mixture was then concentrated under vacuum to remove iPrOH and the mixture was acidified by the addition of 0.1 M HCl until a pH of 3 was reached. The water layer was evaporated under vacuum and the crude product was purified by column chromatography (EtOAc-Hexane 0-100%, then CH_2Cl_2-MeOH 0-3%). Yield: 447 mg (92%). ^{1}H NMR (500 MHz, $CDCl_3$): δ 8.24 (d, J = 8.0 Hz, 2H), 7.45 (d, J = 8.1 Hz, 2H), 2.54 (s, 6H), 2.30 (q, J = 7.5 Hz, 4H), 1.27 (s, 6H), 0.98 (t, J = 7.5 Hz, 6H). ESI-MS(-) *m*/*z* calculated for: $[C_{24}H_{27}BF_2N_2O_2\text{-}H]^-$: 423.21, found: 423.23.

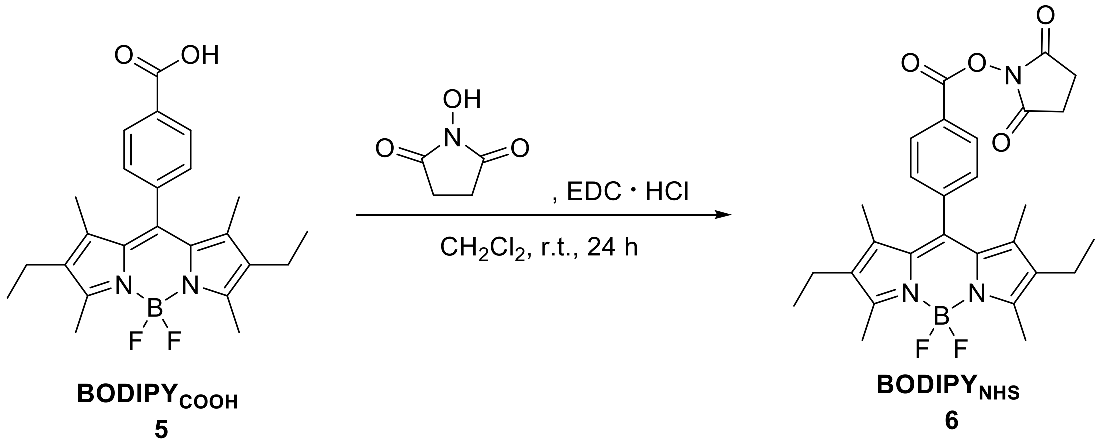

BODIPY$_{COOH}$
5

BODIPY$_{NHS}$
6

2,5-Dioxopyrrolidin-1-yl 4-(2,8-diethyl-5,5-difluoro-1,3,7,9-tetramethyl-5H-5H-4λ^4,5λ^4-dipyrrolo[1,2-c:2',1'-f][1,3,2]diazaborinin-10-yl)benzoate (BODIPY$_{NHS}$ 6).

A 25 mL round bottom was charged with BODIPY$_{COOH}$ (150 mg, 1 eq., 0.35 mmol), *N*-hydroxysuccinimide (122 mg, 3 eq., 1.06 mmol) and 50 mL of CH_2Cl_2 and cooled to 0 °C in an ice bath. 1-(3-Dimethylaminopropyl)-3-ethylcarbodiimide hydrochloride (136 mg, 2.0 eq., 0.71 mmol) was added and the reaction was left to stir overnight at room temperature. The reaction poured into a separatory funnel and washed with water (2x, 50 mL), brine (50 mL), dried with Na_2SO_4, filtered, and dry loaded onto silica. The crude product was purified by column chromatography (EtOAc-hexane 0-50%) to yield an orange-red solid. Yield: 154 mg (83%). ^{1}H NMR (500 MHz, $CDCl_3$): δ 8.26 (d, J = 8.3 Hz, 2H), 7.49 (d, J = 8.3 Hz, 2H), 2.95 (s, 4H), 2.53 (s, 6H), 2.30 (q, J = 7.6 Hz, 4H), 1.27 (s, 6H), 0.98 (t, J = 7.6 Hz, 6H). ESI-MS(+) *m/z* calculated for: $[C_{28}H_{30}BF_2N_3O_4 + H]^+$: 522.23, found: 522.29.

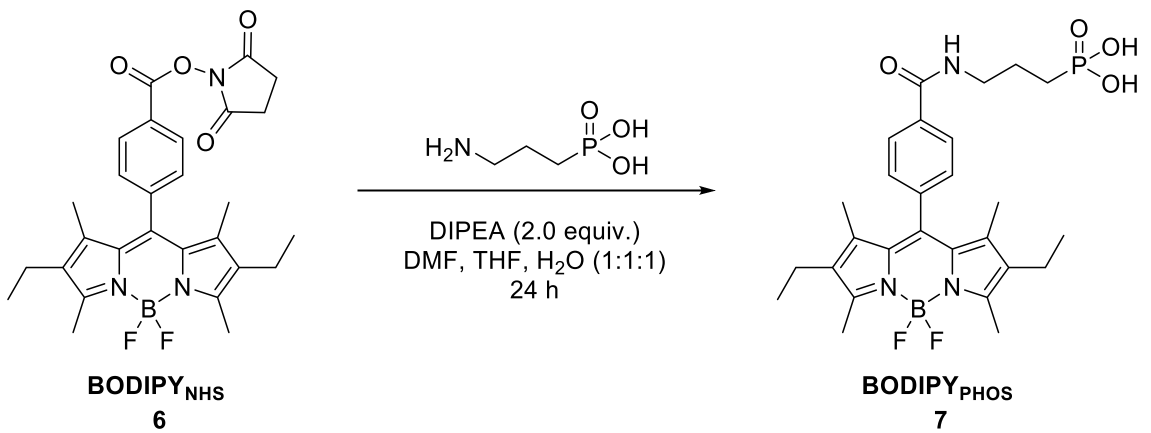

(3-(4-(2,8-Diethyl-5,5-difluoro-1,3,7,9-tetramethyl-5H-5H-4λ^4,5λ^4-dipyrrolo[1,2-c:2',1'-f][1,3,2]diazaborinin-10-yl)benzamido)propyl)phosphonic acid (BODIPY$_{PHOS}$ 7). A 25 mL round bottom was charged with BODIPY$_{NHS}$ (**6**) (37 mg, 1 eq., 71 µmol) and dissolved in 2 mL of dry DMF and 2 mL of THF and cooled to 0 °C in an ice bath. Separately, 3-aminopropylphosphonic acid (15 mg. 1.5 eq., 0.11 mmol) was dissolved in 2 mL of water and DIPEA (25 µL, 2.0 eq., 0.14 mmol) was added. After briefly mixing, the aqueous solution was added dropwise to the DMF/THF solution via syringe and left to stir for 24 h at room temperature. To purify the ligated dye, the reaction was first concentrated under rotary evaporation, then dried under high vacuum with heating at 40 °C for several hours. The residue was then dissolved in DMF, filtered through a plug of celite to remove salts, and purified by reverse phase column chromatography (ACN:H_2O 0.1% formic acid, 20-100%, elutes at 58%) to give the phosphonate dye as a red powder upon removal of solvent. Yield: 33 mg (85%). ^{1}H NMR (500 MHz, DMSO-D_6): δ 8.76 (s, 1H), 8.01 (d, J = 6.9 Hz, 2H), 7.43 (d, J = 8.1 Hz, 2H), 3.30 (d, J = 6.5 Hz, 2H), 2.40 (s, 6H), 2.24 (q, J = 7.8 Hz, 4H), 1.78 – 1.67 (m, 2H), 1.61 – 1.51 (m, 2H), 1.20 (d, J = 2.2 Hz, 6H), 0.89 (t, J = 2.1 Hz, 6H). ^{31}P NMR (202 MHz, DMSO-D_6): δ 26.98. ESI-MS(+) m/z calculated for: $[C_{27}H_{35}BF_2N_3O_4P+H]^+$: 546.25, found: 546.25.

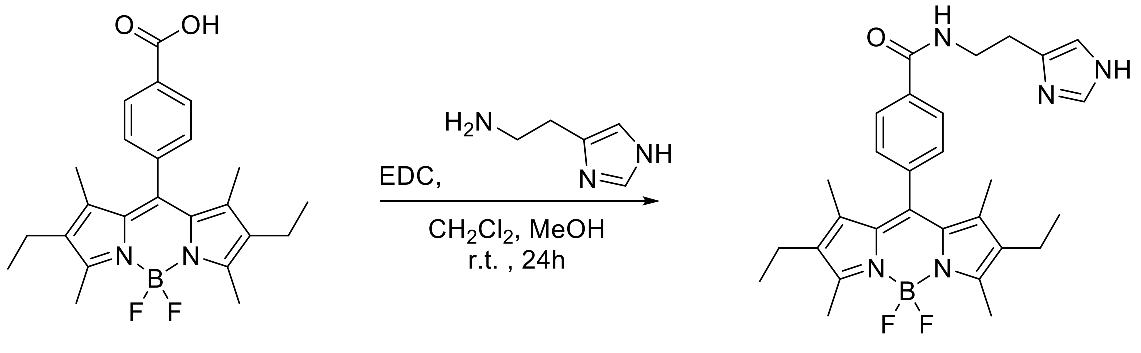

BODIPY$_{COOH}$
5

BODIPY$_{Im}$
8

***N*-(2-(1H-Imidazol-4-yl)ethyl)-4-(2,8-diethyl-5,5-difluoro-1,3,7,9-tetramethyl-5H-4λ^4,5λ^4-dipyrrolo[1,2-c:2',1'-f][1,3,2]diazaborinin-10-yl)benzamide (BODIPY$_{Im}$ 8).** BODIPY$_{COOH}$ (**5**, 20 mg, 0.15 mmol, 1 eq.) was dissolved in 1 mL CH_2Cl_2. Histamine (15 mg, 0.30 mmol, 2 eq.) was dissolved in 500 µL MeOH and added to the CH_2Cl_2 solution, after which EDC (22 mg, 0.30 mmol, 2 eq.) was added and the reaction was left to stir overnight. The reaction mixture was diluted with CH_2Cl_2, dry loaded on silica, and purified by column chromatography (CH_2Cl_2:MeOH 0-6%). Yield: 12 mg (64%). ^{1}H NMR (500 MHz, MeOD-D_4): δ 7.94 (d, J = 8.3 Hz, 2H), 7.59 (s, 1H), 7.35 (d, J = 8.3 Hz, 2H), 6.85 (s, 1H), 3.62 (t, J = 7.3 Hz, 2H), 2.90 (t, J = 7.3 Hz, 2H), 2.47 (s, 6H), 2.26 (q, J = 7.5 Hz, 4H), 1.23 (s, 6H), 0.94 (t, J = 7.6 Hz, 6H). ESI-MS(+) m/z calculated for: $[C_{29}H_{34}BF_2N_5O+H]^+$: 518.29, found: 518.46.

MOF Synthesis

UiO-66. UiO-66 was prepared using a continuous addition method as previously reported (*Angew. Chem. Int. Ed.* **2018**, *57*, 7836-7840). The synthesis of UiO-66_x (x = the particle edge length in nm measured by SEM) at 5 L scale was carried out at 120 °C under atmospheric pressure in DMF using formic acid as a modulator. Two separate 30 mM stock solutions were prepared in 5 L jars. The terephthalic acid (H_2bdc) solution was prepared using 22.5 g of H_2bdc, 4.05 L of DMF, and 450 mL of formic acid, while the $ZrOCl_2{\cdot}8H_2O$ was prepared using 45 g of $ZrOCl_2{\cdot}8H_2O$ in 4.5 L of DMF. The reaction procedure was as follows: 100 mL of the $ZrOCl_2{\cdot}8H_2O$ solution was added to a 5 L round bottom flask at 120 °C, followed by both the $ZrOCl_2{\cdot}8H_2O$ and H_2bdc stock solution delivered separately by peristaltic pump with a feed rate of 12 mL/min for 5 min. The feed rate was accelerated to 32 mL/min for 55 min. After this first addition, 2.5 L of the reaction solution was removed from the reactor to obtain the first product, UiO-66_{80}. Another 1.5 L of metal stock solution and 1.5 L of ligand stock solution were further added to the remaining reaction mixture at 30 mL/min for 50 min. Then 3 L of reaction mixture was collected form the reactor to obtain the second product, UiO-66_{120}. Finally, 1.55 L of metal stock solution and 1.55 L of ligand stock solution were added into the reactor within 1 h at 25.8 mL/min, and the remaining reaction solution (3.7 L) was collected as the third product UiO-66_{250}. All products were first centrifuged (8000 rpm, 30-60 min) and washed with 40 mL DMF twice, and then solvent exchange was performed with by washing 3 times in 40 mL of MeOH. The MOFs were left suspended in MeOH at ~20 mg/mL until further use. Before any experiment, a fraction of the sample was removed and dried to determine the exact weight percent of the suspended particles. For this study, only the

last fraction of UiO-66 particles (UiO-66_{250}) were used. For PXRD and N_2 sorption experiments the samples were dried in vacuum at 120 °C for 24 h. BET surface area: 981 m^2/g.

ZIF-8. ZIF-8 was prepared as previously reported (*J. Am. Chem. Soc.* **2019**, *141*, 51, 20000–20003). Zinc acetate dihydrate (1.50 g, 6.8 mmol) dissolved in 25 mL water was added to 2-methylimidazole (5.30 g, 65 mmol) dissolved in 25 mL of 0.54 mM cetyltrimethylammonium bromide (CTAB) aqueous solution with gentle stirring for 1 min. The mixture turned white after 15 sec and was left undisturbed at room temperature for 2 h. The resulting ZIF-8 particles were collected by centrifugation (fixed-angle rotor, 9,000 rpm, 10 min), washed with 3×40 mL portions of MeOH. To prevent aggregation, the particles were kept suspended in methanol without drying. BET surface area: 1260 m^2/g.

MIL-88B-NH_2. MIL-88B-NH_2 was prepared as previously reported (*Chem. Sci.* **2020**, *11*, 8433-8437). A 150 mL flask with a stir bar was charged with 640 mg of Pluronic F127 surfactant dissolved in 60 mL of deionized water. Iron(III) chloride hexahydrate (716 mg, 2.6 mmol, 2 eq.) was added and the solution was stirred for 1 h, after which acetic acid (2.4 mL, 42 mmol, 32 eq.) was added. After stirring 1 h, 2-aminoterephthalic acid (240 mg, 1.3 mmol, 1 eq.) was added and the suspension was stirred for 2 h. The reaction mixture was transferred to a 100 mL autoclave and placed in a preheated oven at 110 °C oven for 24 h. The solution was cooled to room temperature and the dark brown particles were washed through repeated dispersion/centrifugation cycles with ethanol (4×30 mL, 30 min) and DMF (2×30 mL, 30 min) and left suspended in DMF to prevent particle

aggregation. BET surface area: 13 m^2/g. The low surface area of MIL-88B-NH_2 is a result of the flexibility of this framework, which is in the closed form when dry (*Cryst. Growth Des.* **2013**, *13*, 2286-2291).

<u>Dye Functionalization of MOF Particles</u>

UiO-66 with $BODIPY_{COOH}$. A 100 mL round bottom flask was charged with a stir bar, 400 mg UiO-66, and 50 mL of DMF. Under rapid stirring, 10 mL of a 1 mg/mL solution of $BODIPY_{COOH}$ dissolved in DMF was added dropwise to the UiO-66 suspension and the solution was left to stir for 24 h. The particles were collected by centrifugation (10,000 rpm, 10 min) and washed with acetone (1×80 mL) by repeated centrifugation and resuspension steps (10,000 rpm, 10 min) and left suspended in 80 mL acetone at a concentration of 5 mg/mL for the next experiments.

Functionalization of MIL-88B-NH_2 with $BODIPY_{COOH}$. A 100 mL round bottom flask was charged with a stir bar, 400 mg of MIL-88B-NH_2 and 50 mL of DMF. Under rapid stirring, 10 mL of a 1 mg/mL solution of $BODIPY_{COOH}$ dissolved in DMF was added dropwise to the MIL-88B-NH_2 suspension and the solution was left to stir for 24 h. The particles were collected by centrifugation (10,000 rpm, 10 min.) and washed with acetone by repeated centrifugation and resuspension steps (40 mL, 10,000 rpm, 10 min) and left suspended in 80 mL acetone at a concentration of 5 mg/mL for the next experiments.

Functionalization of ZIF-8 with $BODIPY_{Im}$. A 100 mL round bottom flask was charged with stir bar, 400 mg ZIF-8, and 40 mL of DMF. Under rapid stirring, 25 mg of $BODIPY_{Im}$ was added to the ZIF-8 suspension and the solution was left to stir for 24 h. The particles

were collected by centrifugation (10,000 rpm, 10 min.) and washed with acetone (1x80 mL) by repeated centrifugation and resuspension steps (10,000 rpm, 10 min) and left suspended in 80 mL acetone at a concentration of 5 mg/mL for the next experiments.

Solvent Stability

1.7 mL Eppendorf tubes were charged with 1 mL of solvent and 0.2 mL of a 5 mg/mL suspension of BODIPY-functionalized MOF particles in acetone. Separate tubes were made for each time point: t = 0, 1 h, 24 h. After the specified waiting time, tubes were centrifuged at 13,000 RPM for 10 min and the supernatant was removed for UV-visible spectroscopy. Each time point was performed in triplicate.

Competitive Binding

Stock solutions were prepared by dissolution of each ligand in acetone and serial dilution. Eppendorf tubes (1.7 mL) were charged with 1 mL of ligand solution and 0.2 mL of a 5 mg/mL suspension of BODIPY-functionalized MOF particles in acetone. Points between serial dilutions were created by adding 0.5 mL of ligand solution and 0.5 mL of acetone or 0.25 mL of ligand solution and 0.75 mL of acetone instead of 1 mL of ligand solution. The tubes were allowed to sit for 24 h, followed by centrifugation at 13,000 RPM for 15 min. The supernatant was removed for UV-visible spectroscopy. Each data point was performed in triplicate.

Curve Fitting and Normalization

The sigmoidal binding curves were fit to the logistic equation using the OriginLab logistic fitting function:

$$y = A_{max} + \frac{A_{min} - A_{max}}{1 + \left(\frac{x}{x_0}\right)^p}$$

A_{max} denotes the maximum absorbance, A_{min} denotes the minimum absorbance, x_0 is the concentration at the center of the curve, and p is the slope. To normalize the curves for visualization, the maximum and minimum values were set to 1 and 0, respectively, while keeping x_0 and p the same.

Supporting Figures and Tables

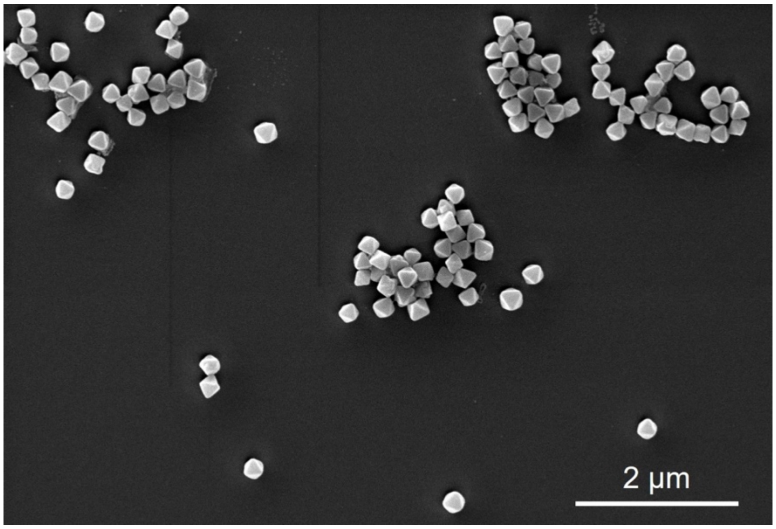

Figure S1. Scanning electron microscopy (SEM) images of UiO-66 particles. The average edge length measured by SEM was 220 nm.

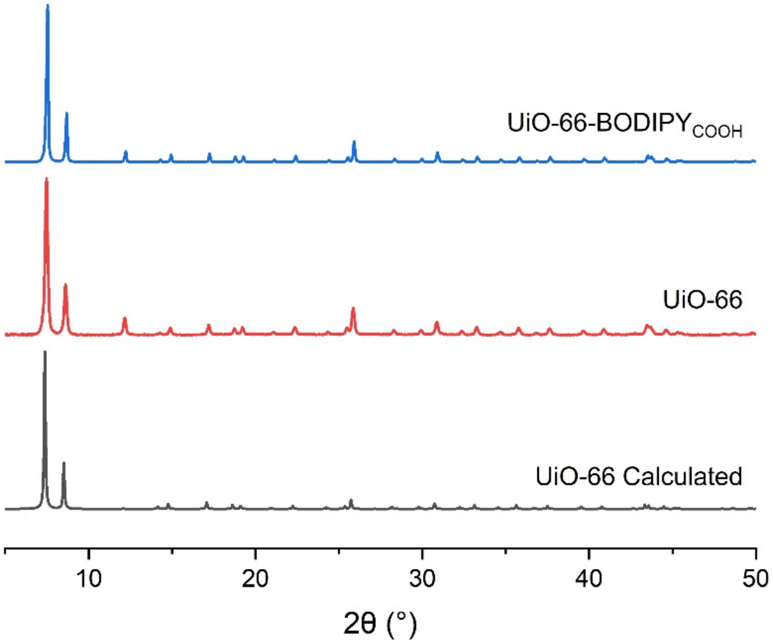

Figure S2. Powder X-ray diffraction (PXRD) of UiO-66 before and after functionalization with BODIPY$_{COOH}$.

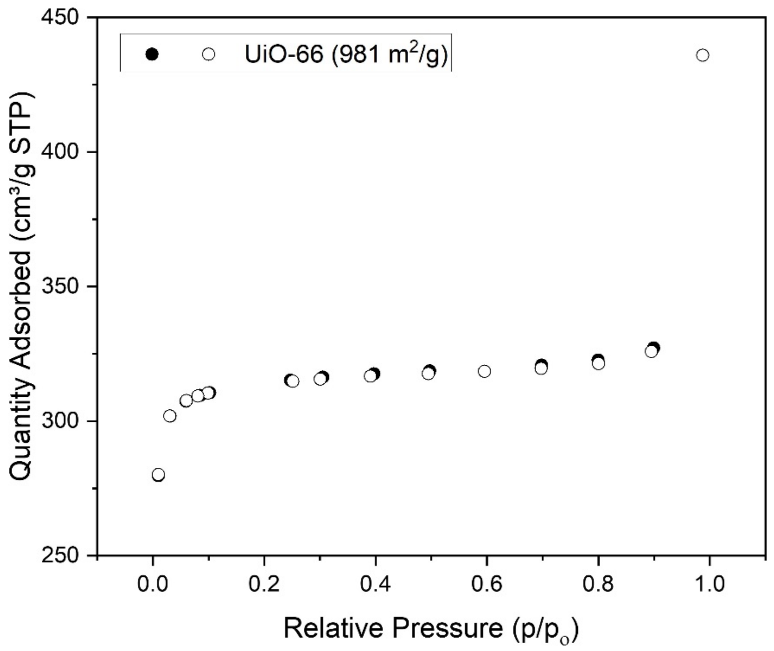

Figure S3. N_2 sorption isotherm for UiO-66 with respective BET surface area.

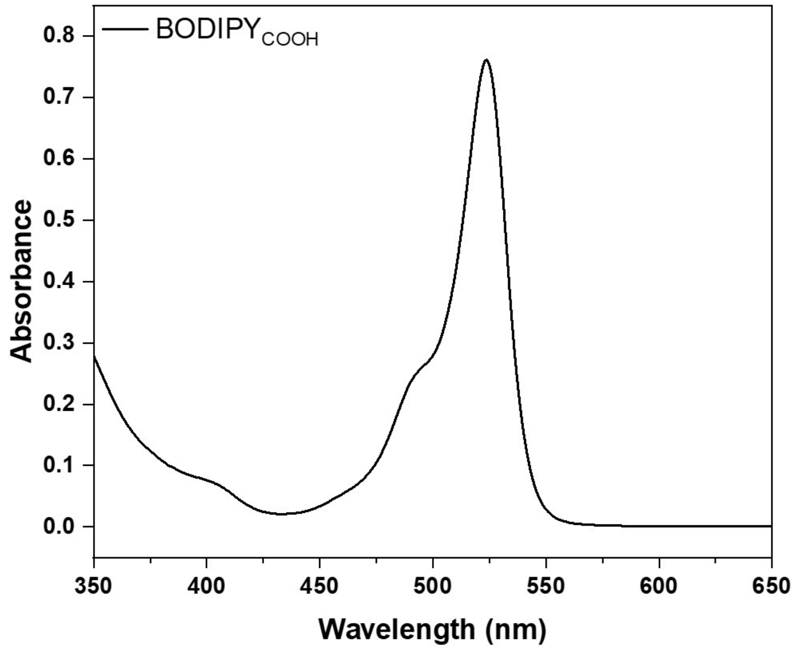

Figure S4. UV-visible spectrum of $BODIPY_{COOH}$ in acetone (λ_{max} = 524 nm).

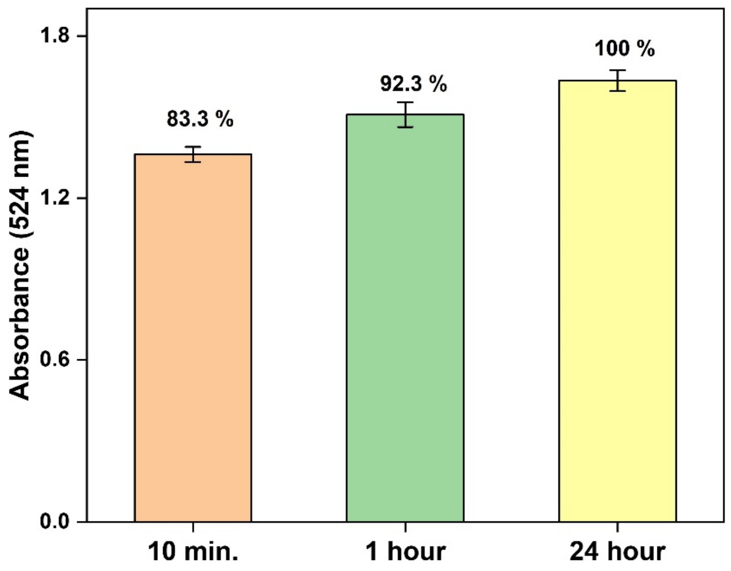

Figure S5. Absorption intensity (524 nm) of the supernatant of digested UiO-66-BODIPY$_{COOH}$ particles isolated at 10 min, 1 h, and 24 h after addition of BODIPY$_{COOH}$. Percentage labels are relative to the absorbance of the 24 h sample representing maximum functionalization.

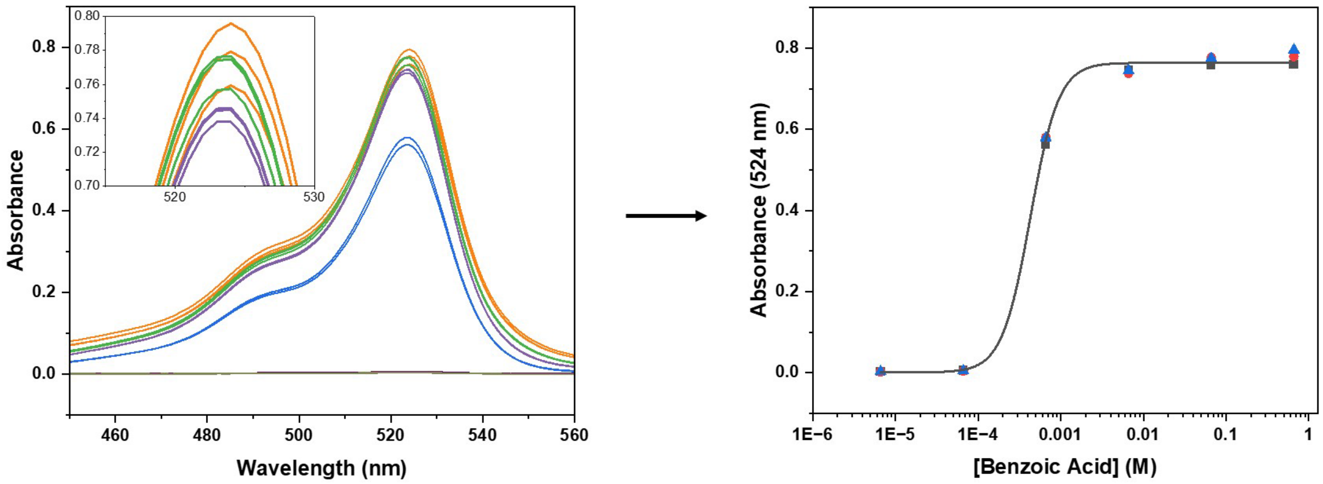

Figure S6. Absorbance (*left*) and sigmoidal curve fitting (*right*) after treating UiO-66-BODIPY$_{COOH}$ with increasing concentrations of benzoic acid (**BA**).

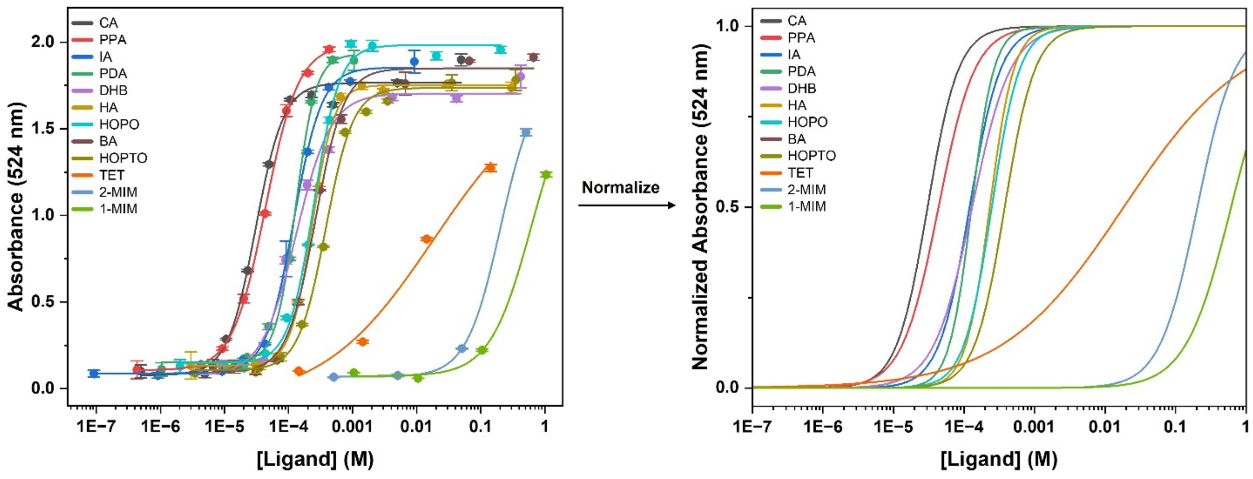

Figure S7. Titration of UiO-66-BODIPY$_{COOH}$ with various ligands. Raw sigmoidal curve fittings with data points (*left*) and normalized curves (*right*).

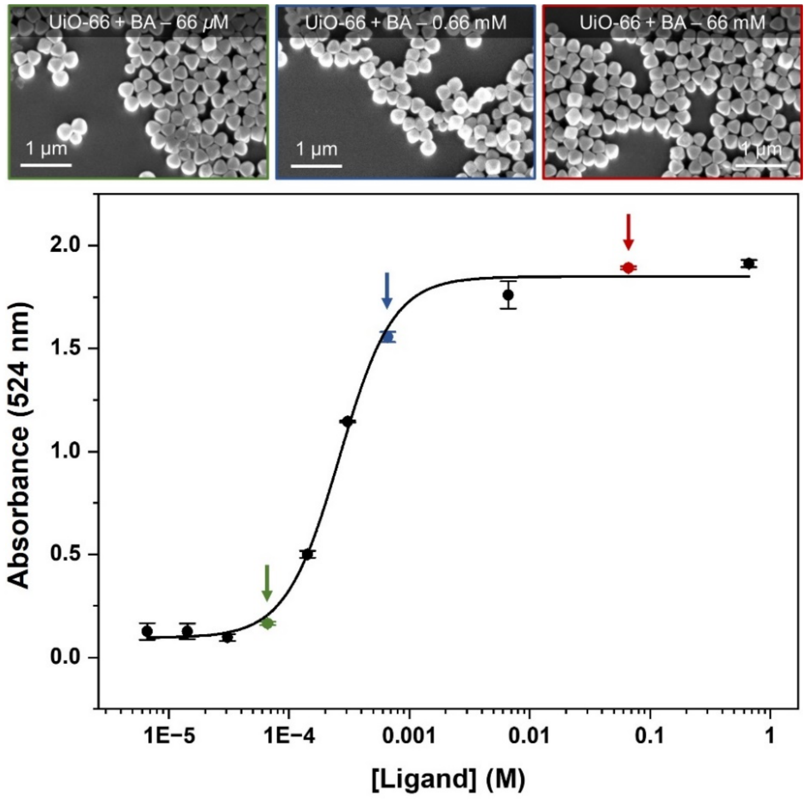

Figure S8. SEM images of UiO-66-BODIPY$_{COOH}$ (*top*) showing no change in particle morphology after titration with benzoic acid (**BA**) at three different concentrations (*bottom*).

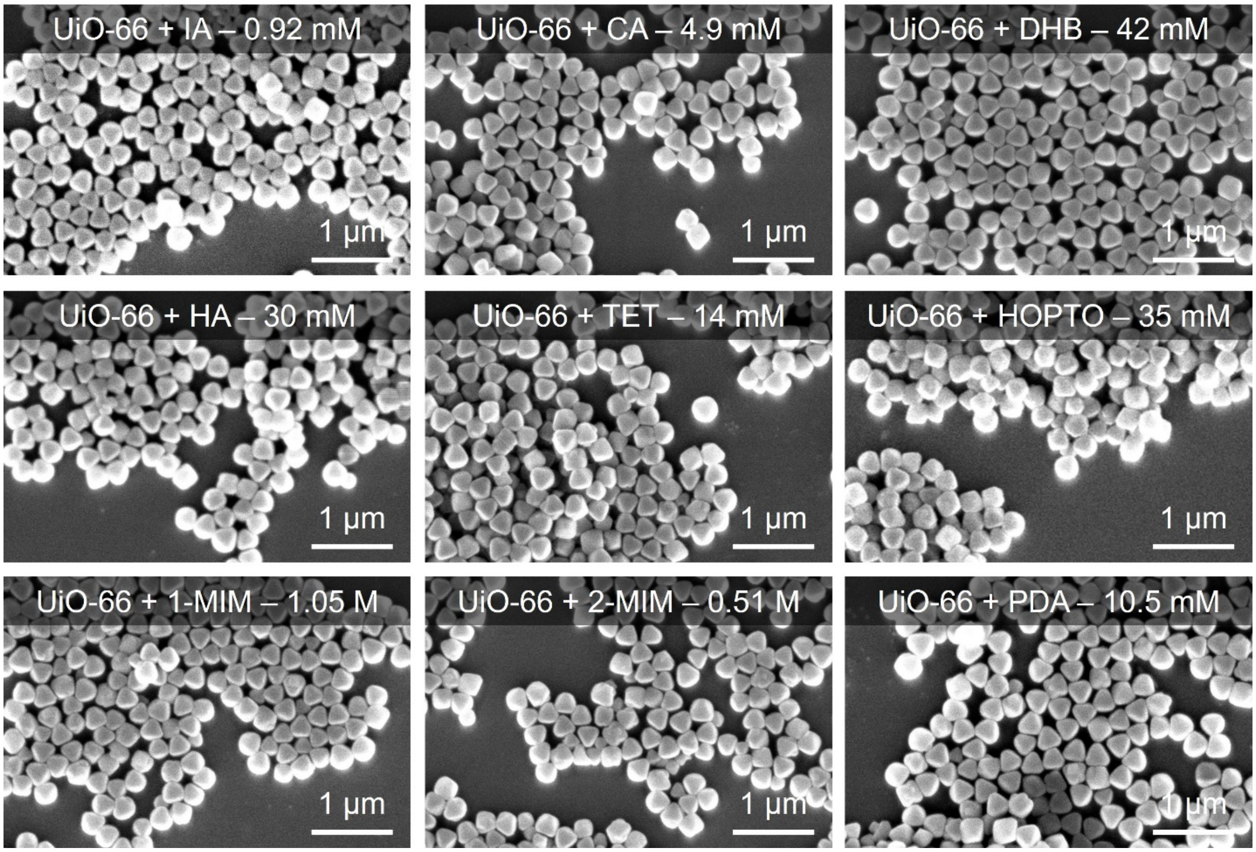

Figure S9. SEM images of UiO-66-BODIPY$_{COOH}$ showing no change in appearance after titration experiments with various ligands. For each ligand, the concentrations listed are above the dye displacement region in Figure S7.

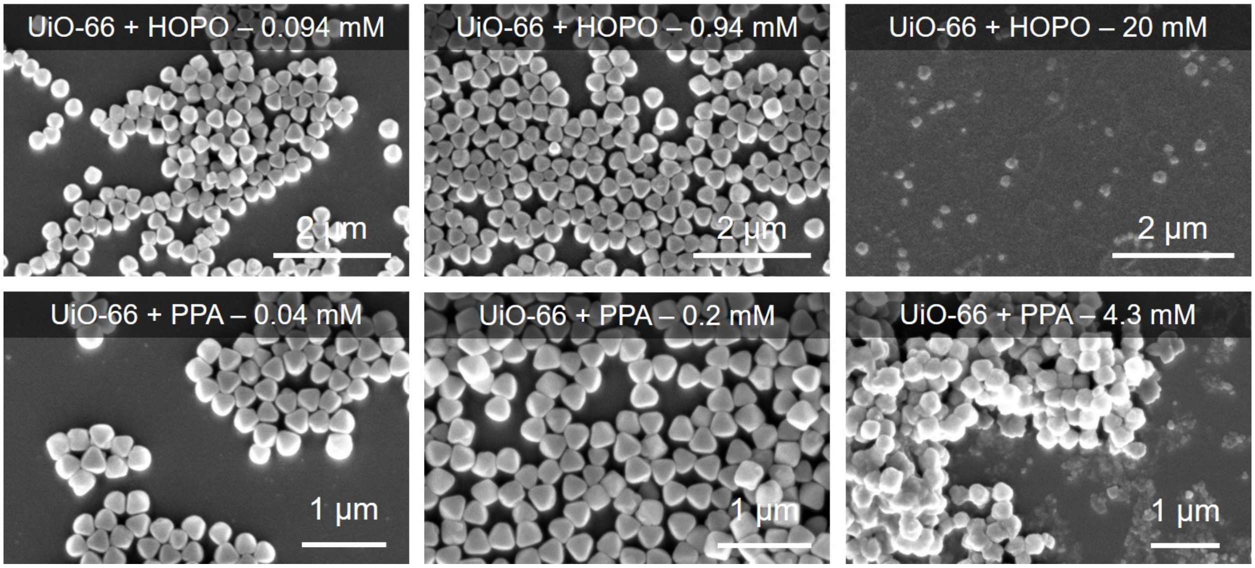

Figure S10. SEM images of UiO-66-BODIPY$_{COOH}$ at various points in the titration with HOPO and PPA showing: particle stability at intermediate dye displacement concentrations of ligand (*left*), concentrations near complete dye displacement (*middle*), and particle degradation at very high concentrations of ligand (*right*). See Figure S8 for highlighted data points along the titration curve.

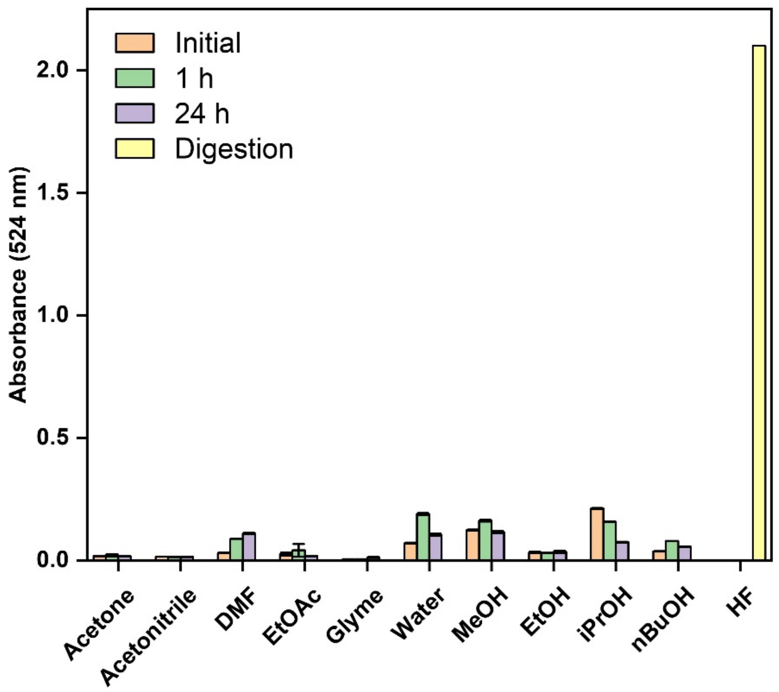

Figure S11. Absorption intensity (524 nm) of the supernatant at three time points from UiO-66-BODIPY$_{PHOS}$ suspended in various solvents.

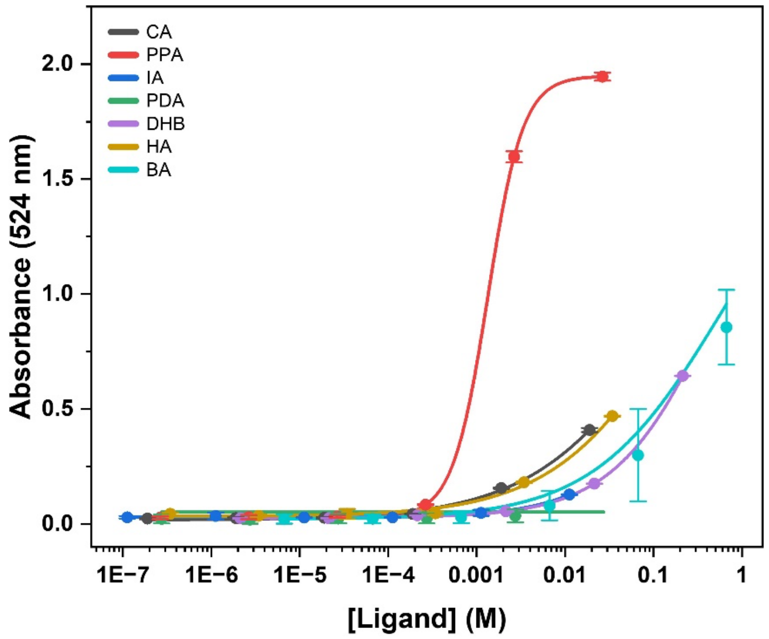

Figure S12. Titration of UiO-66-BODIPY$_{PHOS}$ with select ligands. In the case of **PPA**, the concentrations at which absorbance is detected results in degraded of the UiO-66 particles.

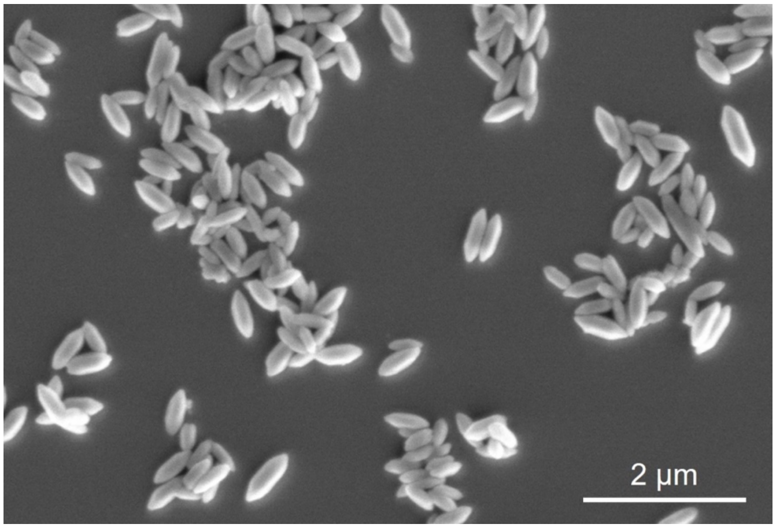

Figure S13. SEM images of MIL-88B-NH_2 particles after synthesis.

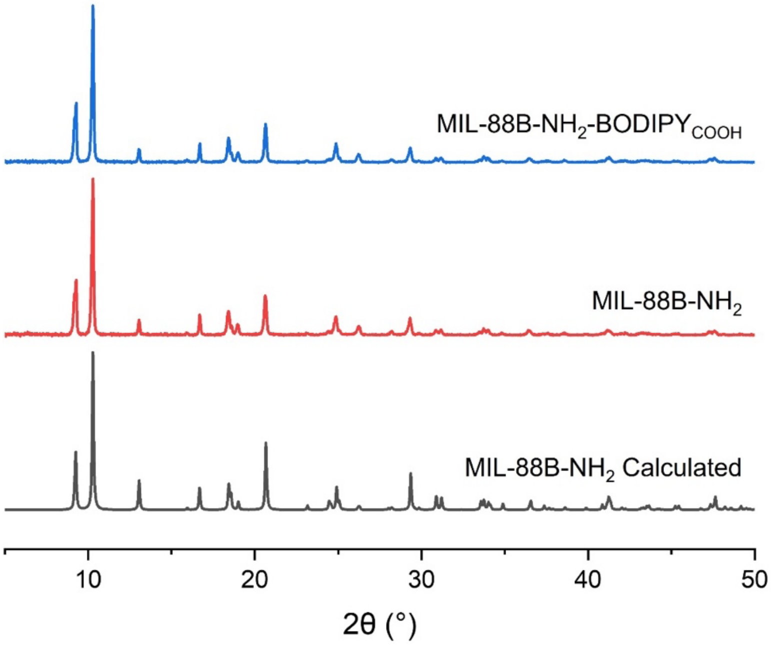

Figure S14. PXRD of MIL-88B-NH_2 before and after functionalization with $BODIPY_{COOH}$.

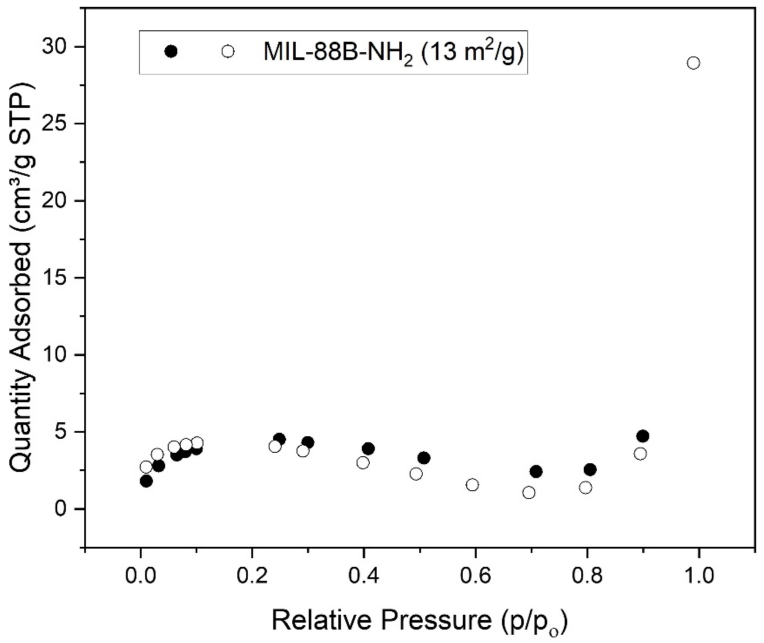

Figure S15. N_2 sorption isotherm for MIL-88B-NH_2 with respective BET surface area.

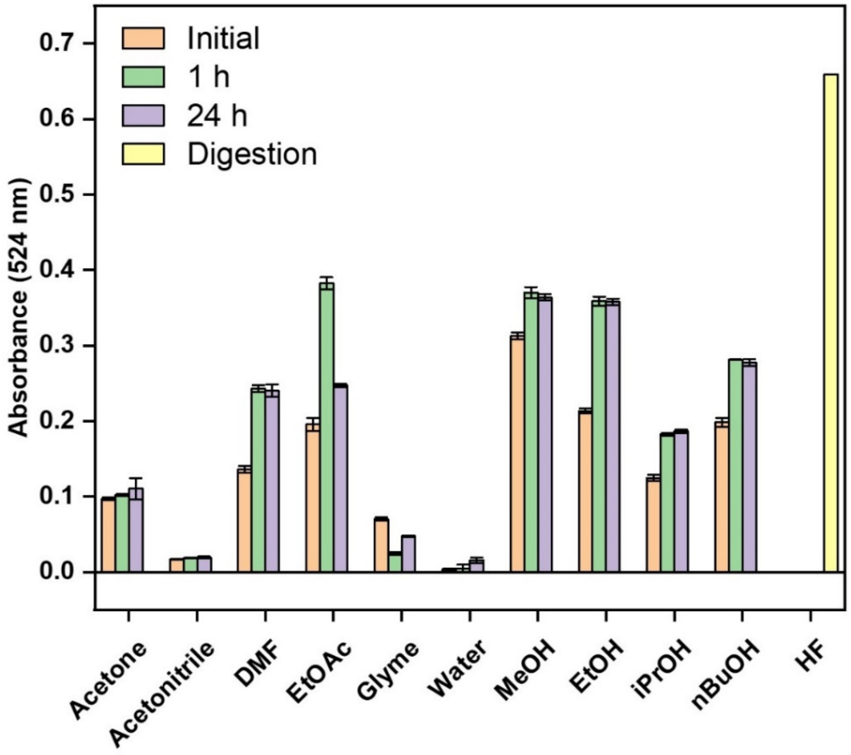

Figure S16. Absorption intensity (524 nm) of the supernatant at three time points from MIL-88B-NH_2-$BODIPY_{COOH}$ suspended in various solvents.

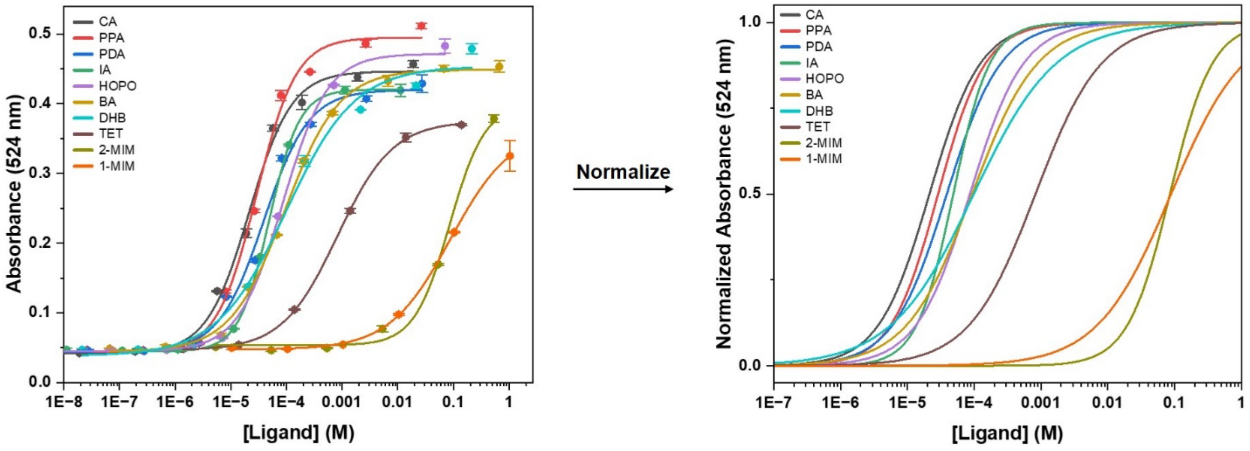

Figure S17. Titration of MIL-88B-NH_2-$BODIPY_{COOH}$ with various ligands. Raw sigmoidal curve fittings with data points (*left*) and normalized curves (*right*).

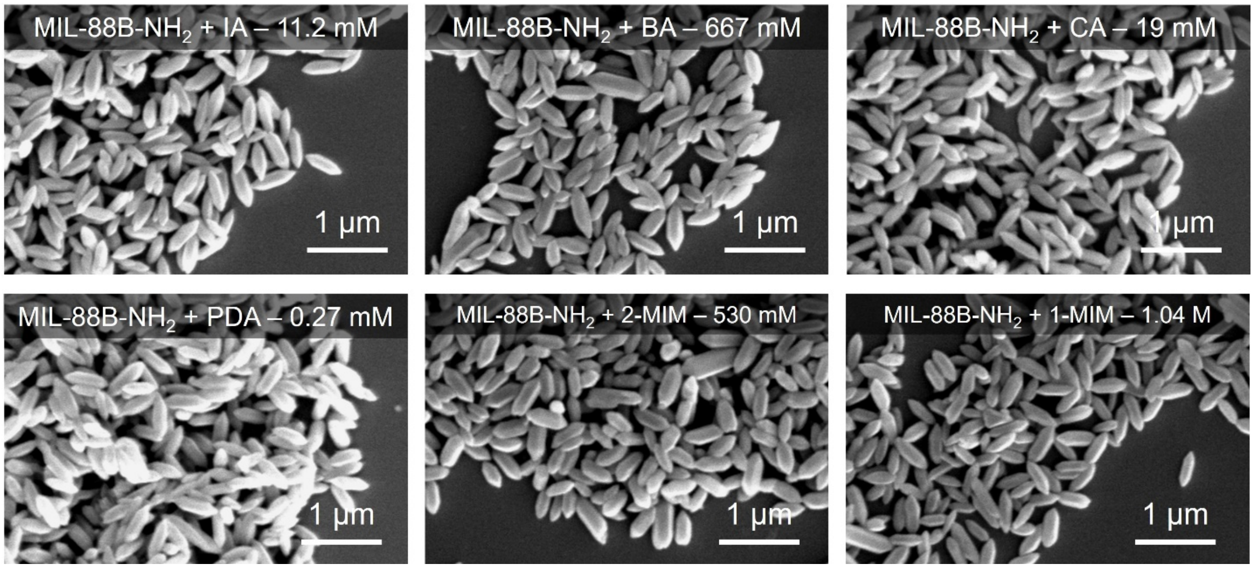

Figure S18. SEM images of MIL-88B-NH_2-$BODIPY_{COOH}$ showing no change in morphology after titration experiments with various ligands. For each ligand, the concentrations listed are beyond the dye displacement region in Figure S17.

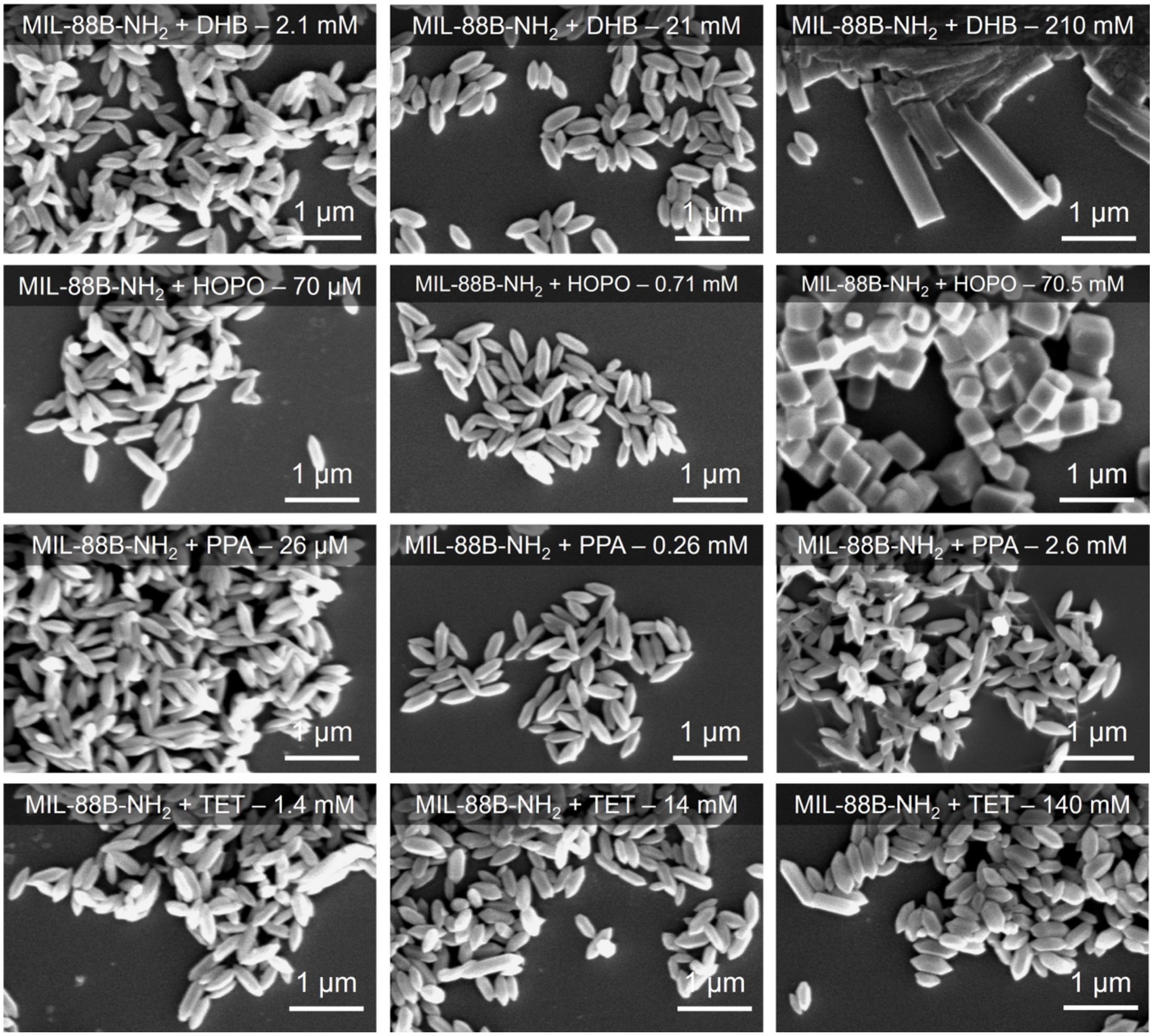

Figure S19. SEM images of MIL-88B-NH_2-BODIPY$_{COOH}$ at various points in the titration with different ligands showing: particle stability at intermediate dye displacement concentrations of ligand (*left*), concentrations near complete dye displacement (*middle*), and particle degradation at very high concentrations of ligand (*right*).

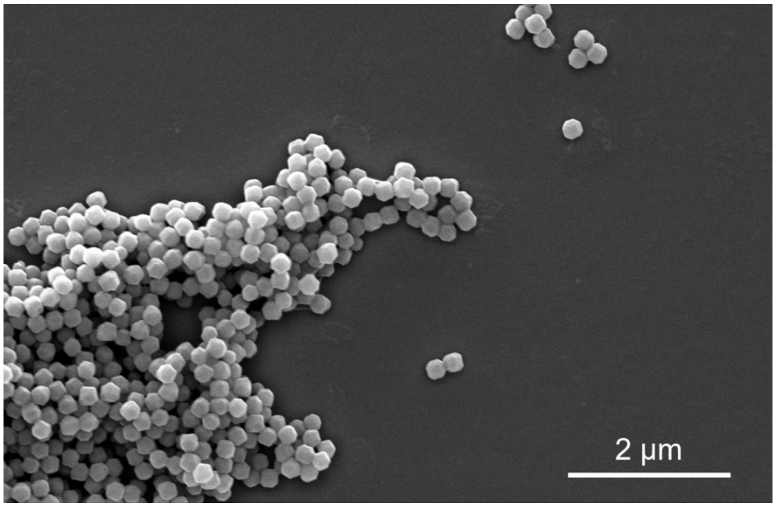

Figure S20. SEM images of ZIF-8 particles after synthesis.

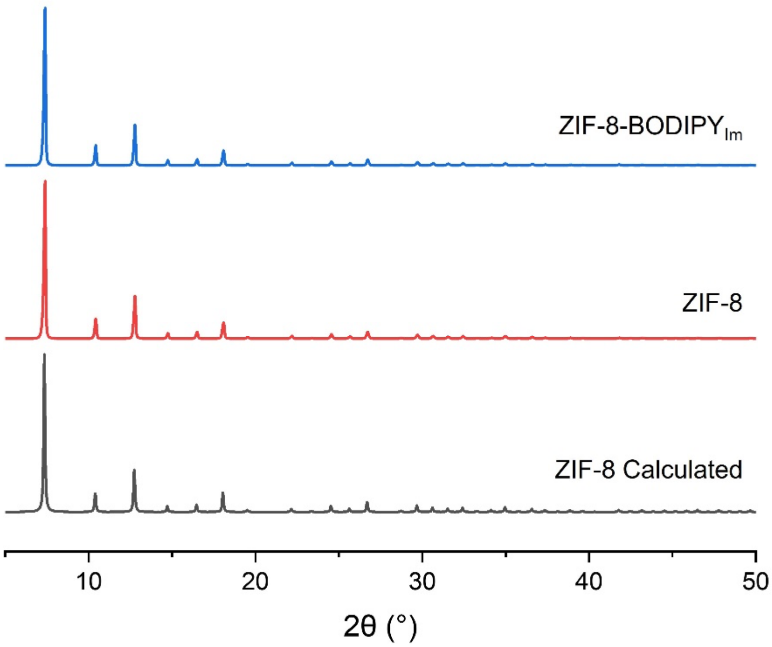

Figure S21. PXRD of ZIF-8 before and after functionalization with BODIPY$_{Im}$.

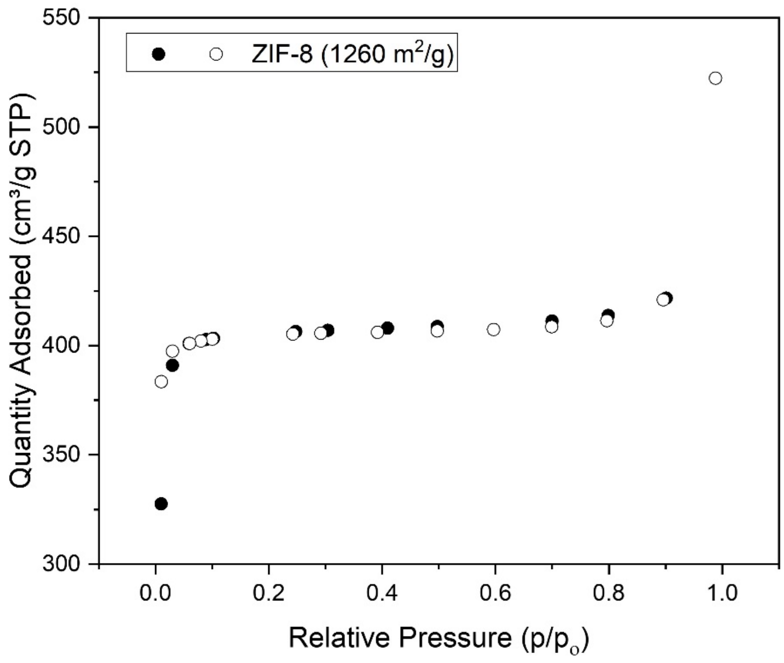

Figure S22. N_2 sorption isotherm for ZIF-8 with respective BET surface area.

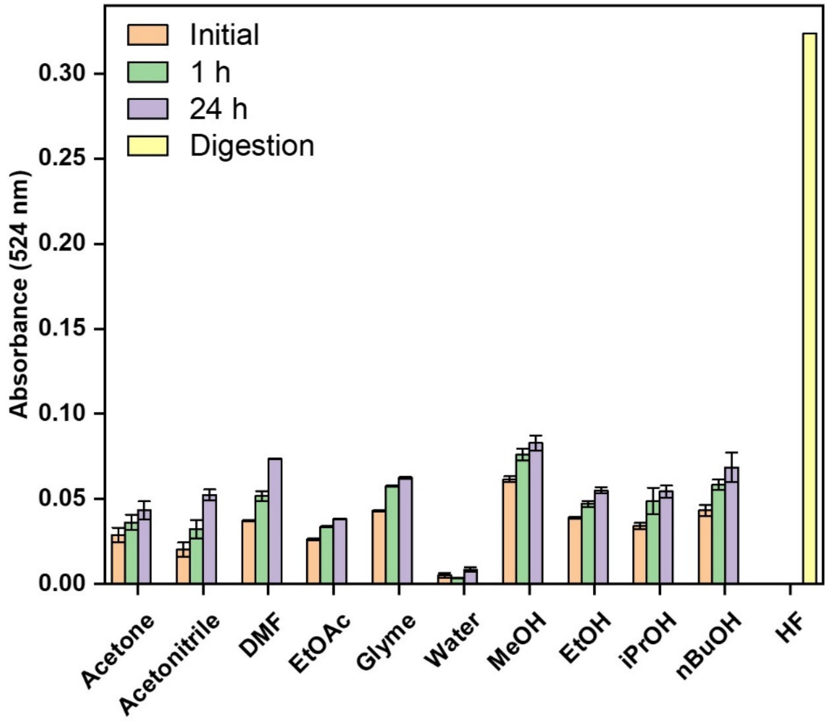

Figure S23. Absorption intensity (524 nm) of supernatant at three time points from ZIF-8-BODIPY$_{Im}$ suspended in various solvents.

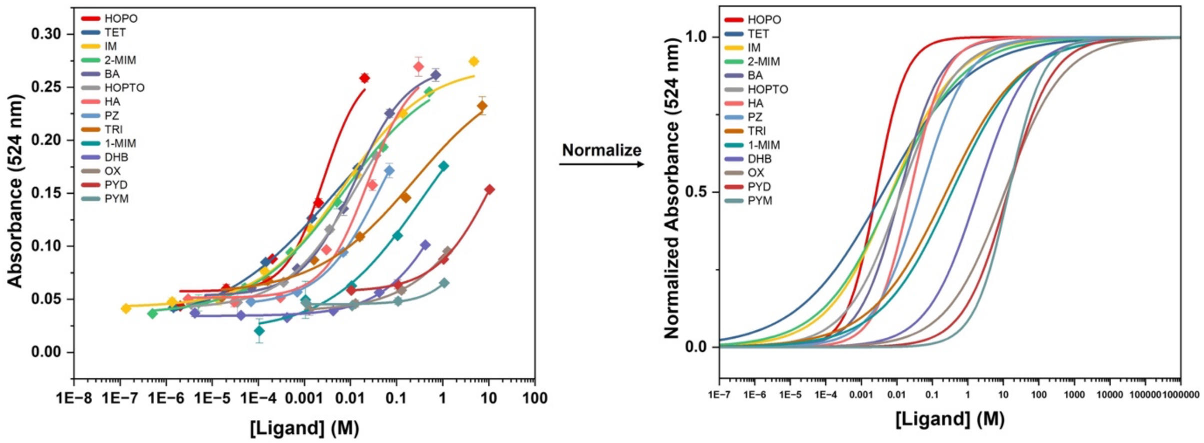

Figure S24. Titration of ZIF-8-BODIPY$_{Im}$ with various ligands. Raw sigmoidal curve fittings with data points (*left*) and normalized curves (*right*).

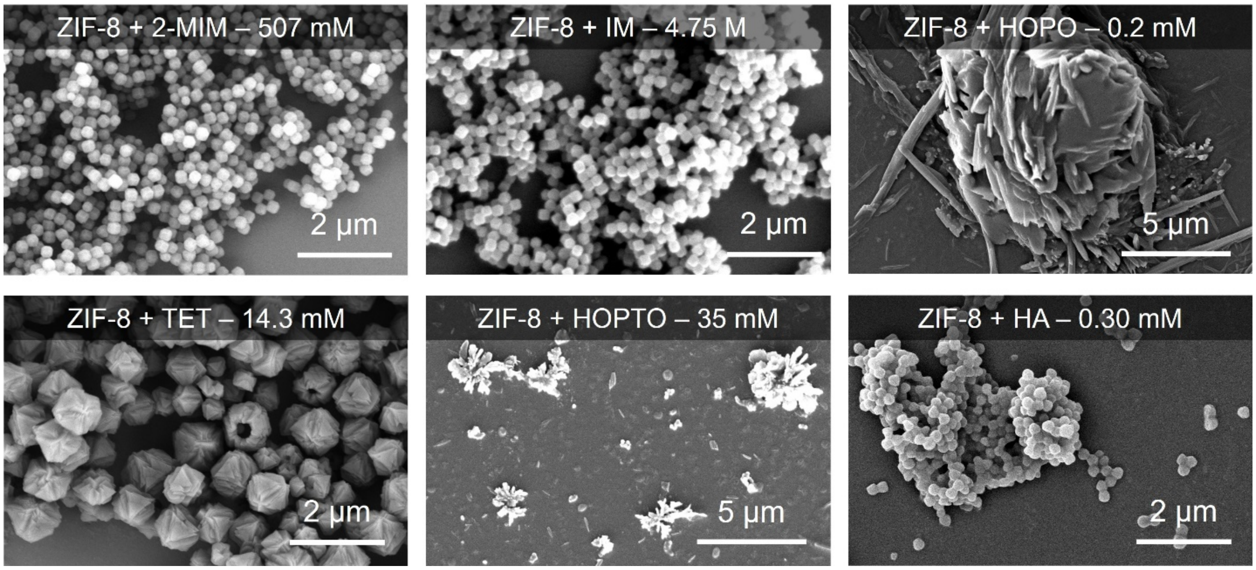

Figure S25. SEM images of ZIF-8-BODIPY$_{Im}$ after titration with select ligands showing stability with **2-MIM** and **IM** and degradation with **HOPO**, **TET**, **HOPTO**, and **HA**.

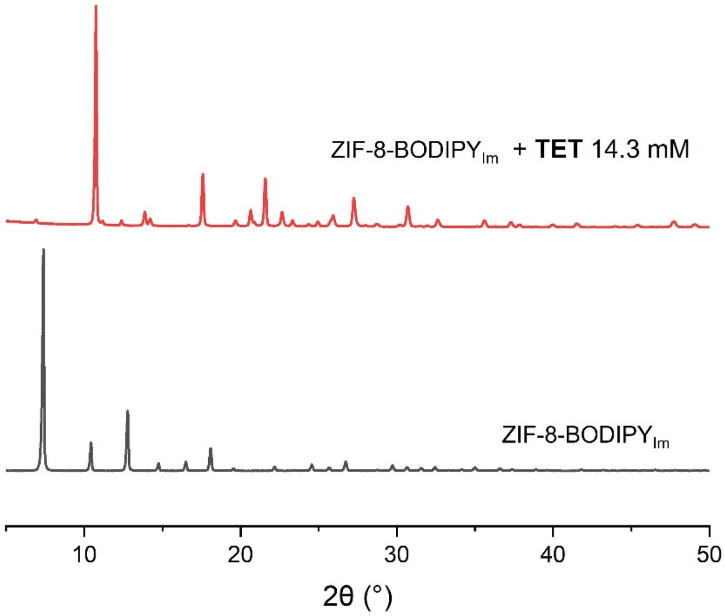

Figure S26. PXRD of ZIF-8-BODIPY$_{Im}$ before and after treatment with 14.3 mM **TET** in acetone.

Ligand	K_{ap} (M^{-1})	Error (±)
HOPO	380	54
TET	245	28
IM	143	18
2-MIM	134	16
BA	80	6
HOPTO	75	8
HA	42	7
PZ	21	1
TRI	4.1	0.8
1-MIM	2.8	0.7
DHB	<1	-
OX	<1	-
PYD	<1	-
PYM	<1	-

Table S1. Table showing ligand abbreviation, apparent binding constant (K_{ap}), and error values from Figure S24. Because the titration curves in Figure S24 were largely incomplete, the values listed here should only be considered useful for relative comparison of affinity.
